# Identification of the proteolytic signature in CVB3-infected cells

**DOI:** 10.1128/jvi.00498-24

**Published:** 2024-07-02

**Authors:** Marli Vlok, Nestor Solis, Jibin Sadasivan, Yasir Mohamud, Reid Warsaba, Jayachandran Kizhakkedathu, Honglin Luo, Christopher M. Overall, Eric Jan

**Affiliations:** 1 Department of Biochemistry and Molecular Biology, Life Sciences Institute, University of British Columbia, Vancouver, British Columbia, Canada; 2 Department of Oral and Biological Sciences, University of British Columbia, Vancouver, British Columbia, Canada; 3 Department of Pathology and Laboratory Medicine, University of British Columbia, Vancouver, British Columbia, Canada; 4 Centre for Heart and Lung Innovation, University of British Columbia, Vancouver, British Columbia, Canada; 5 St. Paul's Hospital, University of British Columbia, Vancouver, British Columbia, Canada; 6 Yonsei Frontier Lab, Yonsei University, Seoul, Republic of Korea; University of Michigan Medical School, Ann Arbor, Michigan, USA

**Keywords:** N-terminomics, degradomics, picornavirus, protease, AIMP2, EMD

## Abstract

Coxsackievirus B3 (CVB3) encodes proteinases that are essential for processing of the translated viral polyprotein. Viral proteinases also target host proteins to manipulate cellular processes and evade innate antiviral responses to promote replication and infection. While some host protein substrates of the CVB3 3C and 2A cysteine proteinases have been identified, the full repertoire of targets is not known. Here, we utilize an unbiased quantitative proteomics-based approach termed terminal amine isotopic labeling of substrates (TAILS) to conduct a global analysis of CVB3 protease-generated N-terminal peptides in both human HeLa and mouse cardiomyocyte (HL-1) cell lines infected with CVB3. We identified >800 proteins that are cleaved in CVB3-infected HeLa and HL-1 cells including the viral polyprotein, known substrates of viral 3C proteinase such as PABP, DDX58, and HNRNPs M, K, and D and novel cellular proteins. Network and GO-term analysis showed an enrichment in biological processes including immune response and activation, RNA processing, and lipid metabolism. We validated a subset of candidate substrates that are cleaved under CVB3 infection and some are direct targets of 3C proteinase *in vitro*. Moreover, depletion of a subset of TAILS-identified target proteins decreased viral yield. Characterization of two target proteins showed that expression of 3C^pro^-targeted cleaved fragments of emerin and aminoacyl-tRNA synthetase complex-interacting multifunctional protein 2 modulated autophagy and the nuclear factor kappa-light-chain-enhancer of activated B cells (NF-κB) pathway, respectively. The comprehensive identification of host proteins targeted during virus infection provides insights into the cellular pathways manipulated to facilitate infection.

**IMPORTANCE:**

RNA viruses encode proteases that are responsible for processing viral proteins into their mature form. Viral proteases also target and cleave host cellular proteins; however, the full catalog of these target proteins is incomplete. We use a technique called terminal amine isotopic labeling of substrates (TAILS), an N-terminomics to identify host proteins that are cleaved under virus infection. We identify hundreds of cellular proteins that are cleaved under infection, some of which are targeted directly by viral protease. Revealing these target proteins provides insights into the host cellular pathways and antiviral signaling factors that are modulated to promote virus infection and potentially leading to virus-induced pathogenesis.

## INTRODUCTION

Viral myocarditis affects all age groups, ethnicities, and sexes—approximately 22 per 100,000 people, with a median age of 42 years (1, 2). Viral infection can lead to inflammation of the myocardium, a symptom that can develop into dilated cardiomyopathy (DCM). Up to 15% of viral infected patients develop acute myocarditis during their illness and ~20% of patients with myocarditis, confirmed by histology, will develop DCM resulting in cardiac enlargement and congestive heart failure (3–5). Up to 60% of patients with myocarditis and DCM exhibit virus infections of the heart (6, 7). Despite the severe consequences of these viral infections, no effective treatments currently exist.

One of the main culprits causing viral myocarditis is the endemic coxsackievirus B3 (CVB3), the exemplar isolate of the species *Enterovirus B*, one of 12 enterovirus (EV-A to EV-L) and 3 rhinovirus (RV-A to RV-C) species in the genus *Enterovirus* (family *Picornaviridae*, subfamily *Ensavirinae*) [ICTV (ictvonline.org)]. *Enterovirus B* is the most detected species of enterovirus worldwide and is highly prevalent on all continents (8). Viruses of the genus *Enterovirus* are well-established causative agents of human diseases, including common cold, diarrhea, hand-foot-mouth disease, conjunctivitis, encephalomeningitis, paralysis, and diabetes. Despite the severity of enteroviral infection, there are no effective treatments, and the only prophylactics are the FDA-approved vaccines for poliovirus (PV) and an enterovirus A71 vaccine licensed by the China National Medical Products Administration (9). The lack of medical treatment highlights the importance of revealing virus-host interactions of enteroviruses that may highlight new antiviral strategies.

CVB3 contains a ~7.4 kilobase positive-sense RNA genome encoding a single polyprotein, the translation of which is facilitated by an internal ribosome entry site (IRES). After translation, the polyprotein is cleaved by two virally encoded proteases, 2A protease (2A^pro^—MEROPS C03.020) and 3C protease (3C^pro^—MEROPS C03.011), resulting in sequential cleavage into P1 (structural proteins), P2 (2ABC), and P3 precursors (3ABCD) and then subsequently processed into the individual, mature viral proteins, four structural proteins (VP1–4), and seven non-structural proteins (2A, 2B, 2C, 3A, 3B, 3C, 3D). 3C^pro^ mediates the majority of cleavage events, while the 2A^pro^ is primarily responsible for excising itself from the upstream VP1 protein (10). An autocatalytic cleavage occurs between VP4 and VP2 likely using an aspartate-dependent type of autocatalytic cleavage at Asp2011 and Asn4069 hydrogen bond (11, 12).

2A^pro^ and 3C^pro^ are cysteine proteases that belong to the chymotrypsin endopeptidase protease family, which uses cysteine as a nucleophile (13, 14). While these proteases share approximately 20% amino acid identity, they have similar tertiary structures with conserved amino acids surrounding the catalytic residues (15). Both proteases have two domains that participate in the formation and positioning of the active site or catalytic triad, which is composed of histidine, aspartic acid (2A^pro^)/glutamic acid (3C^pro^), and the cysteine as the nucleophile (16). 3C^pro^ and its precursor form 3CD^pro^ target the preferred cleavage motif, AXXQ↓GPXX, where X denotes any amino acid and the down arrow represents the scissile bond between the P1′ to P4′ and P1 to P4 residues, respectively (17). The 2A^pro^ cleaves between a tyrosine-glycine separating the P1 structural proteins and non-structural proteins in the initial polyprotein cleavage events (18).

In addition to the processing of the viral polyprotein, both 2A^pro^ and 3C^pro^ also target host cellular proteins, which have been studied since the early 1980s and has revealed key pathways that are modulated and targeted by enterovirus proteases to promote virus infection (15, 19). One of the most studied is the targeting of the eukaryotic translation initiation factor 4 G (eIF4G) by PV 2A^pro^. Cleavage of eIF4G, which is a component of the eIF4F cap-binding complex and promotes ribosome binding to the mRNA, during PV infection results in shutoff of host translation, which in effect blocks the expression of host antiviral factors and increases the pool of available ribosomes for viral protein synthesis (20–22). As such, the PV IRES mechanism can still recruit ribosomes to drive viral protein expression utilizing a subset of translation initiation factors including the cleaved C-terminal fragment of eIF4G. Thus, this viral strategy highlights the modulation of a key host factor by a viral protease that alters host processes (i.e., shutoff of host translation), facilitating viral protein synthesis and infection. Enterovirus proteases target host proteins that modulate a number of cellular processes, including transcription, cytoskeleton, nucleocytoplasmic transport, stress granule formation, and antiviral responses (15, 19). Targeting signaling factors within major innate immune response pathways, such as the mitochondrial antiviral signaling protein (MAVS) by both 2A^pro^ and 3C^pro^ proteases and the Toll-like receptor adaptor molecule 1 (TRIF), 14-3-3epsilon, and retinoic acid-inducible gene 1 by CVB3 3C^pro^, attenuates the host types I and II interferon (IFN) responses, thereby evading the host antiviral responses and allowing virus infection (23–25).

In addition to facilitating the viral life cycle, CVB3-mediated proteolysis has also been linked to disease onset. The cleavage of both dysferlin and dystrophin by 2A^pro^, respectively, disrupts membrane repair and the structure of the sarcolemma, leading to myocarditis and cardiomyopathy, while cleavage of the serum response factor by 2A^pro^, a cardiac-enriched transcription factor, results in downregulation of cardiac-specific contractile and regulatory genes, leading to impaired cardiac function (26–29). Cleavage of nucleoporin 98 under CVB3 infection induces a cascade of events resulting in an impaired cardioprotective NRG1 signaling pathway (30). Given the links to proteolysis, the identification of other host cellular proteins that are targeted by both viral and cellular proteases may shed light into the pathogenesis mediated by enterovirus infection.

Recent proteomic, specifically, N-terminomic approaches have accelerated the unbiased identification of protein substrates of viral proteases (31–35). Terminal amino isotopic labeling of substrates (TAILS) identifies natural N- and neo-N-termini peptides using a negative selection approach and has been successful in identifying the protein targets of both cellular and viral proteases (32, 35–39). Protease-generated N terminus (neo-N-terminus) peptides are isolated by negative selection and then identified by tandem mass spectrometry (MS/MS). TAILS is sensitive, quantitative, and simultaneously identifies substrate and cleavage sites, thus providing functional insights to *in vivo* pathways. We previously used TAILS to identify host cellular substrates of PV 3C^pro^ using an *in vitro* cleavage assay, revealing high-confidence functionally relevant substrates (32, 40). In this study, we used TAILS for the first time to identify host cellular proteins that are cleaved in virus-infected cells. Applying this approach to two different cell lines, we identified and validated numerous host proteins targeted for cleavage under CVB3 infection and a subset of proteins targeted by CVB3 3C^pro^ *in vitro*. Functional analysis of two targets, emerin (EMD) and aminoacyl-tRNA synthetase complex-interacting multifunctional protein 2 (AIMP2), using knockdown and overexpression approaches revealed the importance of these proteins in the virus replication cycle. This study further highlights the utility of the N-terminomic TAILS approach to reveal the proteomic signatures in pathogen-infected cells.

## MATERIALS AND METHODS

### Cell culture and virus stocks

HeLa and HEK293 cells were cultured in Dulbecco’s modified Eagle medium (DMEM), and HL-1 murine cardiac muscle cells were cultured in Claycomb medium (Sigma, Cat #51800C) with an additional 2 mM L-glutamine and 0.1 mM norepinephrine in ascorbic acid. All cell culture media were supplemented with 10% fetal bovine serum and 1% penicillin-streptomycin and cultured at 37°C. CVB3 (Kandolf strain; GenBank accession number M33854.1) and PV (Mahoney type 1 strain; GenBank accession number NC_002058.3) were propagated and titered in HeLa cells.

### Virus infections

Virus was absorbed at the specific multiplicity of infection (MOI) in HeLa and HL-1 cells for 30 min and 1 h, respectively, in serum-free DMEM or Claycomb medium at 37°C, washed with phosphate-buffered saline (PBS) at pH 7.4 and replaced with the appropriate complete medium.

Intracellular and extracellular virus titers were measured from virus-infected pellets and cell supernatants, respectively. Cell pellets were prepared by washing cells twice with PBS and lysing by three freeze-thaw cycles. Titers were quantified using plaque assays as previously described (32).

### N-terminal TAILS proteomics

TAILS was performed as previously described (36). Briefly, cells (confluent T75) were washed with PBS three times and resuspended in 500 µL lysis buffer (0.5% SDS, 10 mM dithiothreitol [DTT], 100 mM HEPES, 10 mM EDTA, protease inhibitors, pH 7.5). Cells were lysed and the genomic DNA sheared by tip probe sonication for 30 s on, 60 s off (on ice) for a total of three cycles. Cell debris was removed by centrifugation (12,000 *g* at 4°C for 10 min), supernatants made up to 1 mL with H_2_O and treated with 10 mM DTT for an hour at 37°C. Iodoacetamide was added to a final concentration of 20 mM and incubated at room temperature in the dark for 10 min. Proteins were precipitated with chloroform:methanol and 500 µg of protein was resuspended in 3M GuCl and 100 mM HEPES, pH 8.0, to a final concentration of 1 mg/mL. Protein N-termini were isotopically labeled using 2.5 µL of 1 M heavy (+34 Da) (for virus-infected samples) or light (+28 Da) (for mock-infected samples) formaldehyde and 2.5 µL of 500 mM NaCNBH_3_ overnight at 37°C. Excess formaldehyde was quenched with 50 mM Tris, pH 8.0, final concentration. Samples were mixed, precipitated as before, and resuspended in 600 mM GuCl, 100 mM HEPES, pH 8.0. Proteins were digested with 1 mg/mL sequencing-grade trypsin (Thermo Fisher Scientific) at a ratio of 1:50 protease to substrate at 37°C for 16 h with rotation. For preTAILS, 20 µL of the digested peptide was desalted using C18 StageTips, lyophilized, and stored at −20°C prior to liquid chromatography-tandem mass spectrometry (LC-MS/MS) analysis. The N-terminal peptides in the remaining sample were enriched by depleting tryptic peptides via covalent coupling to dendritic hyperbranched polyglycerol-aldehydes (HPG-ALD) polymer (https://ubc.flintbox.com/technologies/888fc51c-36c0-40dc-a5c9-0f176ba68293) at a 5:1 polymer to peptide (wt:wt) ratio in the presence of 30 mM NaCNBH_3_ for 16 h at 37°C at a pH of 8.0. Excess formaldehyde was quenched with 50 mM Tris, pH 8.0, final concentration. N-terminal-labeled peptides were retrieved by ultrafiltration using 18 kDa filters, desalted using C18 StageTips, lyophilized, and stored at −20°C prior to LC-MS/MS.

Data-dependent acquisition was performed using an Easy nLC-1000 UHPLC (Thermo Fisher Scientific) coupled to an Impact II Q-TOF mass spectrometer (Bruker-Daltonics) with CaptiveSpray nanoBooster ionization interface. One microgram of peptides was injected onto a 75 um × 300 mm analytical column with ReproSil-Pur C18 1.8 µm stationary phase (Dr. Maisch GmbH) and eluted using a 120-min curved gradient at 250 nL/min from 5% to 24% in buffer B (99.9% acetonitrile, 0.1% formic acid), which was increased to 34% over 10 min, and a final increase to 95% buffer B over 5 min which was held for 10 min at 95%. The mass spectrometer was operated in positive ion polarity mode with a CaptiveSpray source voltage of 1,250 V, and precursor ions were detected from 150 to 2 250 m/z. MS/MS spectra were acquired by a Top12 selection method with MS/MS summation time (intensity-adjusted with duty cycle of 1.3–1.8 s). Acetonitrile was used as dopant in the NanoBooster.

### Mass spectrometry data analysis

MS/MS data were analyzed using the Mascot (Matrix Science) MS/MS Ions Search (41), searching against the UniProt human and mouse databases (UP000005640: accessed January 2019; UP000000589: accessed March 2019) that included the CVB3 (Kandolf strain; GenBank accession number M33854.1) genome. Main search parameters included semiArg-C, maximum of 1 missed cleavages, Carbamidomethyl (C) fixed modification, Acetyl (N-term), Dimethyl (K), Dimethyl (N-term), Dimethyl:2H (4)13C(2) (K), and Dimethyl:2H (4)13C(2) (N-term) as variable modifications, peptide tolerance of 20 ppm, MS/MS tolerance of 0.05 Da, a peptide charge of 2+, 3+, and 4+, error tolerant, and decoy search.

Data were further analyzed using Skyline (42, 43). Dimethylation (Ndm) and acetylation (Nother) searches were conducted separately with structural modifications of Carbamidomethyl (C), Dimethyl (K), and Dimethyl (N-term) and isotope label type heavy modifications Dimethyl 13C (2)2H(4) (N-term) and Dimethyl 13C (2)2H(4) (K) for the Ndm search and structural modifications of Carbamidomethyl, Dimethyl (K), and Acetyl (N-term) along with isotope label type heavy modifications Dimethyl 13C (2)2H(4) (K) for Nother. Internal standard type was set as light. A cutoff score of 0.90 was employed and no ambiguous matches were included. Precursor charges of 2, 3, and 4 were included with a mass accuracy of 20 ppm. Further parameters included were Semi-ArgC, C-terminal with semi-cleavage allowed, a minimum of one peptide per protein, and repeated peptides removed.

Quantile-quantile probability (QQ) plots were generated by calculating the Z-scores of the ranked peptides and analyzing them compared to the corrected H/L ratio. Venn diagrams were generated with InteractiVenn (44). Cellular protease cleavage sites were identified using TopFINDER as part of TopFIND4.1 (45)

### Immunoblot analysis

Equal amounts of proteins were resolved by SDS-PAGE analysis and transferred to polyvinylidene difluoride membrane. Antibodies and dilutions used in this study were as follows: EMD D3B9G_XP (1:1,000, Cat #30853), CNOT2 D8Z8 (1:1,000, Cat #34214), fatty acid synthase (FASN) C20G5 (1:1,000, Cat #3180), B-Tubulin (1:1,000, Cat #2146), NUP98 C39A3 (1:1,000, Cat #2598) (Cell Signalling), NUDT21 2203C3 (1:1,000, Cat #sc-81109), PLA2G4A 4-4B-3C (1:1,000, Cat #sc-454), HNRNPH2 1G11 (1:1,000, Cat #sc-32310), HNRNPM M1-4 (1D8) (1:1,000, Cat #sc-20002), TRAF2 H-10 (1:1,000, Cat #sc-7346) (Santa Cruz), LSM14A (1:3,000, Cat #18336-1-AP), AIMP2/JTV-1 (1:1,000, Cat #10424-1-AP), PPP1R13L (1:2,000, Cat #51141-1-AP) (Proteintech), Larp4 (1:2,000) (generously provided by Richard Maraia), VP1 (1:1,000, Cat #M706401-1 Dako and Cat #M47 Mediagnost), ECL anti-Rabbit IgG HRP (1:5,000, Cat #NA934 Amersham), Goat anti-mouse IgG-HRP Na9340v (1:5,000, Cat #sc-2005 Santa Cruz).

### *In vitro* cleavage assay

*In vitro* cleavage assays were performed as previously described using HeLa extracts (32). Briefly, cell extracts were incubated with CVB3 protease 3C^pro^, 2A^pro^, catalytically inactive 3C^pro^ (C147A), and 2A^pro^ (C57A) mutant proteins in cleavage reaction buffer (20 mM HEPES, pH 7.4, 150 mM KOAc, 1 mM DTT) at 37°C for different times (32). Reactions were stopped by addition of Laemmli’s SDS-PAGE sample buffer.

### Plasmids and transfections

The coding sequences for AIMP2, emerin, and enhanced green fluorescent protein (eGFP) were obtained from pBluescript JTV-1 (Addgene #16473), emerin pEGFP-N2 (Addgene #61985), and pEGFP-N (Addgene #6080-1). Full-length, N-terminal, and C-terminal AIMP2 and emerin were cloned into pEGFP-N using Gibson assembly (Cat #E2611 NEB). PCR was conducted as per manufacturer’s protocol using the Phusion high fidelity master mix (Cat #M0531 NEB), with overlapping primers for pEGFP-N3 as the vector and AIMP2 full length, N- and C-termini as the inserts. *Escherichia coli* strain DH5α were transformed with completed Gibson-assembled plasmids, screened, and sequence confirmed.

HeLa cells were transfected with 1.5 to 2 µg of plasmid using Lipofectamine 3000 (Cat #L3000001 Invitrogen) as per manufacturer’s protocol.

### siRNA knockdowns

HeLa cells were transfected with 60 pmol siRNA using Lipofectamine RNAiMAX (Cat #13778100 Invitrogen) as per manufacturer’s protocol for 48 h with the following siRNAs: Ambion Silencer siRNA PPP1R13L ID114674, EMD ID7812, CNOT2 IDs226691, JTV-1 ID126664, and Negative Control No. 1. Knockdown efficiency was validated by immunoblotting, and cells were used for virus infection.

### Compound treatment of CVB3-infected cells

HeLa cells were pre-treated for 30–60 min with cerulenin (45 µM) (with repeated treatment after 1.5 h) (46), N-(p-amylcinnamoyl) anthranilic acid (ACA) (20 µM) (47) or InSolution BAY11-7082 (Calbiochem Sigma-Aldrich) (10 µM) prior to infection (MOI 1.0 or 0.1) and harvested at 4.5 h post infection (h.p.i.).

### NFĸB and interferonβ (IFNβ) reporter assays

HeLa cells were co-transfected with the expression plasmid (1.5 µg), a constitutively expressing Renilla luciferase construct (40 µg) (SV40 promoter), and either an NFĸB-responsive (200 µg) or interferonβ-responsive (300 µg) (IFNβ_pGL3 Addgene #102597) firefly luciferase reporter construct for 12–16 h. Prior to tumor necrosis factor alpha (TNFα—Thermo Fisher Scientific) stimulation (100 ng/µL), cells were starved for 60 min in serum- and antibiotic-free DMEM. TNFα stimulation was conducted in starvation media for 2 h. Five micrograms of polyinosinic:polycytidylic acid high molecular weight (HMW) (InvivoGen) was transfected, and cells were harvested 8 h after treatment. Cells were washed once with PBS and collected in passive lysis buffer (Promega) and stored at −80°C. NFĸB or IFNβ luciferase and control Renilla luciferase activities were measured using the dual-luciferase reporter assay system according to the manufacturer’s protocol (Promega).

### Immunofluorescence

HeLa cells on coverslips were fixed with 10% neutral buffered formalin (MilliporeSigma) for 15 min and washed three times with PBS. Cells were subsequently permeabilized with 0.2% Triton in PBS for 15 min, blocked for 30 min (0.2% Triton X-100, 3% bovine serum albumin [BSA], PBS), and incubated with primary antibody (in blocking solution) for 1 h at room temperature. Antibodies used include EMD D3B9G_XP (1:100, Cat #30853 Cell Signalling), AIMP2/JTV-1 (1:50, Cat #10424–1-AP Proteintech), p65 (1:200, Cat #51-0500 Thermo Fischer Scientific), and VP1 (1:200, Cat #M706401-1 Dako). Cells were washed three times with PBS, followed by secondary antibody (Texas Red Goat anti-rabbit 1:1,000, Cat #T2767, Alexa 488-Goat anti mouse 1:1,000, A21141 Life technologies) incubation in blocking solution for 1 h. Three PBS washes were done before incubating cells for 15 min in PBS containing Hoechst dye (1:20,000), followed by a final PBS wash. Coverslips were mounted using Prolong Gold antifade reagent (Cat #P10144 Life Technologies) and cells were imaged using a Leica SP5 confocal microscope (Leica Microsystems, Wetzlar, Germany) with a 63× objective. Representative images are shown and were analyzed in Image J.

### Statistical analysis and graph generation

Data were plotted and analyzed using GraphPad Prism 9. All data were calculated with 95% confidence intervals unless stated otherwise. Probability values (*P*) were determined using nested *t*- or one-way analysis of variance tests with either Dunnett’s multiple comparisons test or Tukey’s multiple comparisons test. Statistical significance was determined as a *P*-value of <0.05 and is denoted by an asterisk as follows: *P* ≤ 0.05 (*), *P* ≤ 0.01 (**), *P* ≤ 0.001 (***), and *P* ≤ 0.0001 (****).

## RESULTS

### TAILS analysis of CVB3 proteins in infected cells

**Fig 1:**
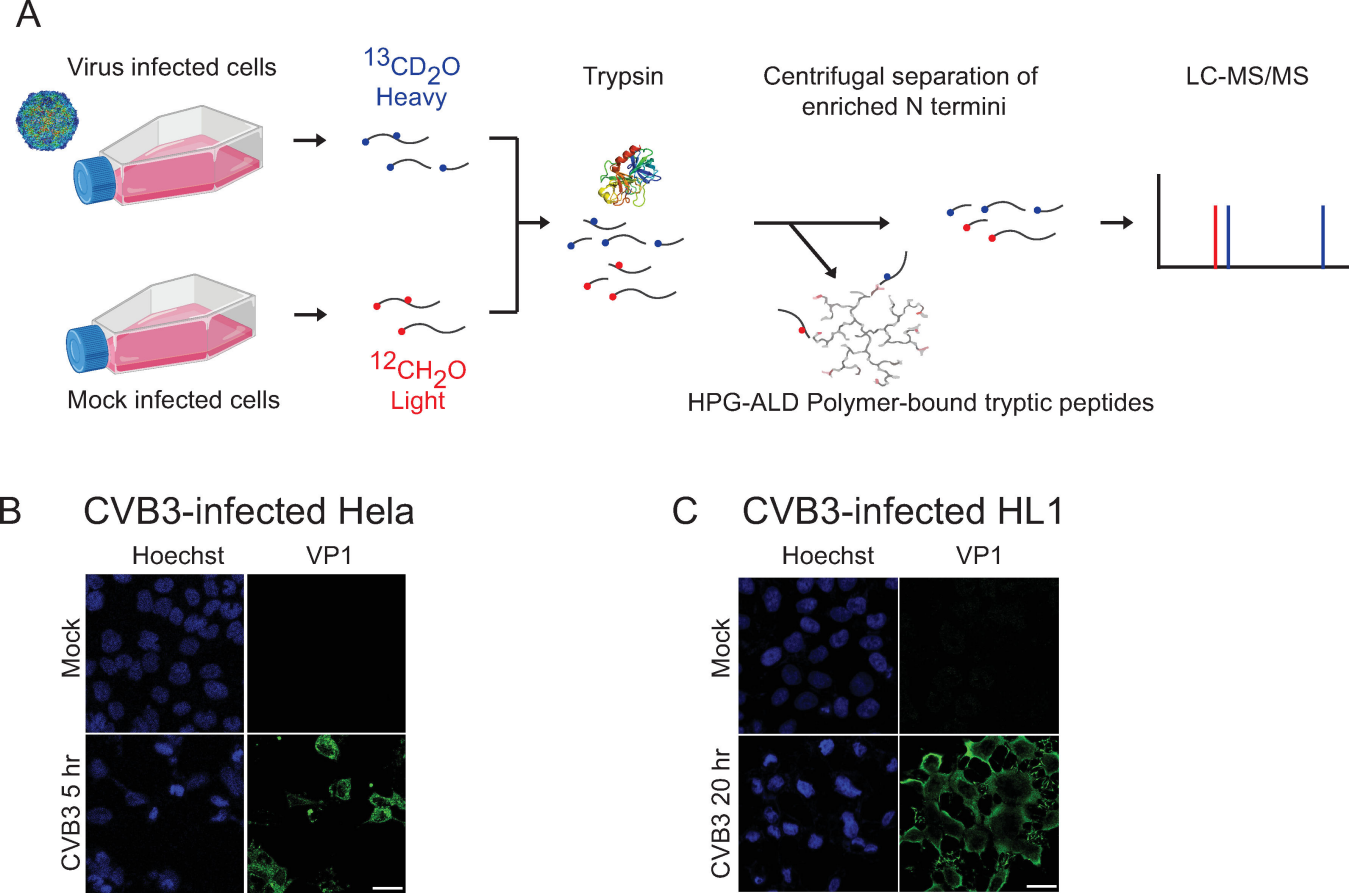
Strategy for identifying proteolytic substrates in CVB3-infected cells using TAILS. (**A**) Flowchart of TAILS approach for CVB3-infected HeLa and HL-1 cells. Immunofluorescence of mock- and CVB3-infected (**B**) HeLa cells (MOI 10, 5 h.p.i., scale bar 20 µm) or (**C**) HL-1 cells (MOI 50, 20 h.p.i., scale bar 10 µm) stained with 4′,6-diamidino-2-phenylindole (DAPI) (blue) and VP1 (green).

We previously identified *bona fide* host protein substrates of the enteroviruses PV and CVB3 3C proteases using an *in vitro* TAILS approach (32). To determine the proteolytic signatures in a more physiological infection model, we performed TAILS in CVB3-infected HeLa and mouse cardiomyocyte HL1 cells. High MOIs resulted in >95% infection of HeLa at 5 h.p.i. and HL1 cells at 20 h.p.i. as monitored by VP1 structural protein antibody immunofluorescence staining (Fig. 1A and B). We showed previously that CVB3 infection of HeLa and HL1 cells led to observed cleavage of known host 3C^pro^ substrates, such as HNRNPM and PABP (32, 40).

Proteins from mock and infected cell lysates were isotopically labeled by reductive dimethylation of primary amines, applying an isotopically heavy (+6 Da) or medium (+4 Da) (defined as H) formaldehyde to the infected protein extracts versus a light (L) formaldehyde to the mock-infected protein samples (Fig. 1A). Corresponding samples were mixed and trypsinized, and trypsin-generated N-terminally unlabeled peptides were removed by their coupling to the dendritic aldehyde polymer. The enriched neo- and natural N-terminus peptides were collected by ultrafiltration from the polymer containing sample and then identified by LC-MS/MS. Two independent experiments (*N* = 2) of CVB3-infected HeLa and HL-1 cells were performed in triplicate (*n* = 3). We identified 14,986 and 14,310 total N-terminal blocked peptides from CVB3-HeLa and CVB3-HLI TAILS, respectively. From these, 5,343 and 4,667 peptides from CVB3-HeLa and CVB3-HLI TAILS, respectively, were N-terminal dimethylated. The fraction of the N-terminal dimethylated peptides that are neo-N-termini peptides ranged between 93% and 96% of total peptides depending on the replicate experiment. From the CVB3-HeLa TAILS, 1,121 N-terminal dimethylated unique peptides were identified from 817 unique proteins, whereas from CVB3-HL1 TAILS, 1,575 N-terminal dimethylated unique peptides were identified from 968 unique proteins (Table S1). Peptides were ranked by isotopic heavy/light (H/L——infected/mock) ratios and QQ plots were used for each biological replicate to determine the high and low statistical isotopic H/L ratio cutoffs (Table S1; Data S1) by selecting peptides outside of the linear range of the data distribution. TAILS identifies N-termini peptides and neo-N-termini peptides; hence, we focused on neo-termini peptides for our analysis and peptides with a positive H/L ratio outside of the linear range (Table S1; Data S1). Finally, candidate selection was prioritized based on cross-referencing the data set with published data on picornavirus immunoprecipitations, comprehensive identification of RNA-binding proteins by mass spectrometry (ChIRP-MS), functional-knockdown screens, and degradomics (31, 32, 48–52).

**Fig 2:**
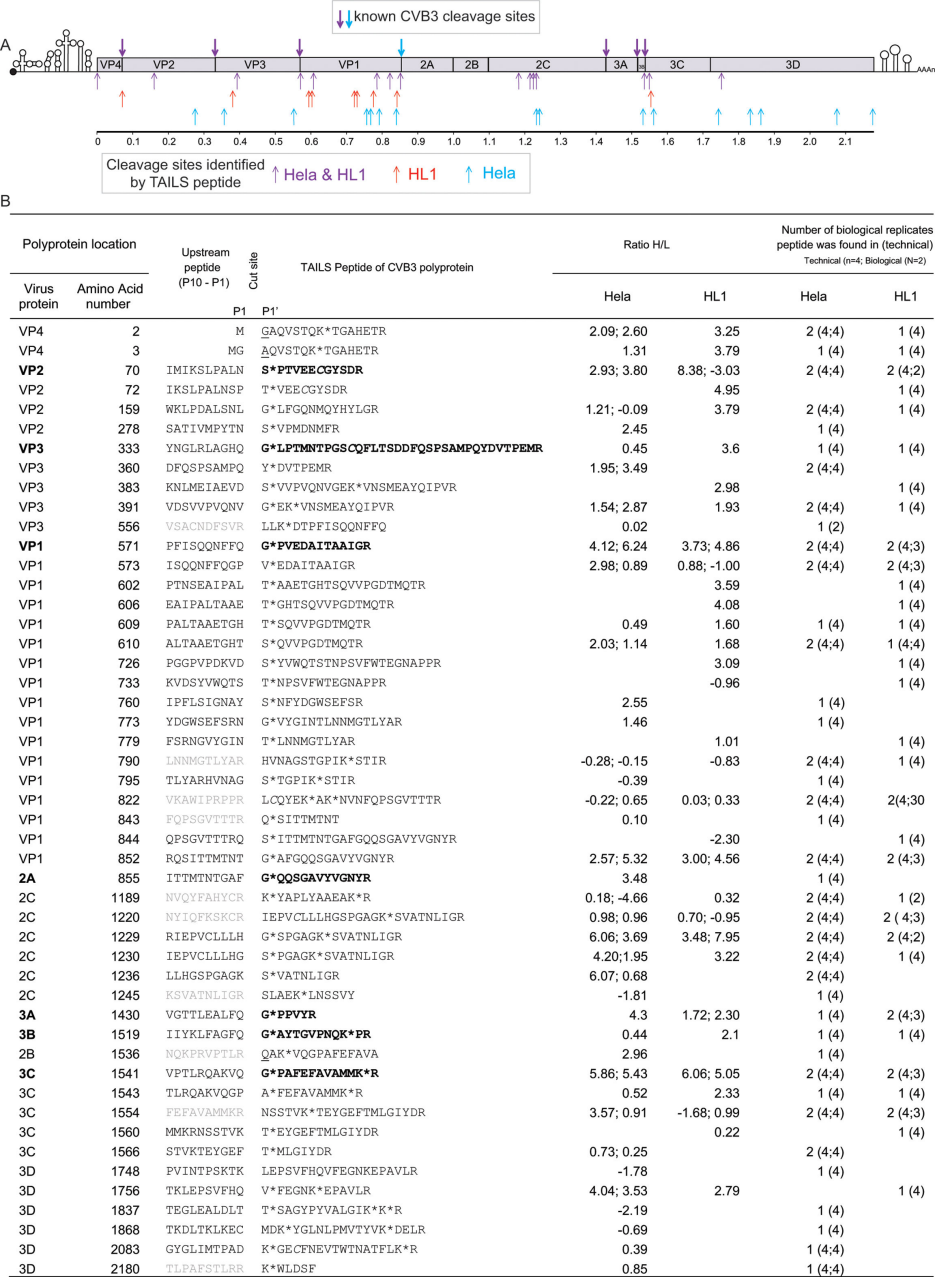
N-terminal peptides of the CVB3 polyprotein detected by TAILS. (A) Graphical presentation of cleavage sites detected in the polyprotein inferred by TAILS analysis, with known cleavage sites indicated above. TAILS analysis also detected other potential cleavage site indicated below. Colors depict data sets in which TAILS peptides were detected: both HeLa and HL-1 (purple), HL-1 (red), and HeLa (blue). (B) Table showing TAILS-identified peptides within the CVB3 polyprotein including the peptide locations and the high-to-low (H/L) ratios in both biological replicates. Number of biological replicates and technical replicates in which the peptides were detected are also included. Peptide modifications are indicated by asterisk (*, dimethylated), underlined (acetylation), and italics (carbamidomethylation). Predicted upstream peptides are included (P10–P1) with those displaying an arginine in P1 in gray. Known CVB3 cleavage peptides are indicated in bold.

We first examined the TAILS-generated peptides that mapped to the CVB3 open reading frame. Peptides were identified throughout the CVB3 polyprotein confirming productive infection. Notably, several neo-N peptides mapped to most of the known 2A^pro^ and 3C^pro^ cleavage sites of the CVB3 polyprotein (Fig. 2A; Data S1). Peptides representing N-terminal cleavages of 2B, 2C, and 3D were not detected and the natural N-terminus of the polyprotein shows evidence of ragging (by exopeptidase activity) and was acetylated. Interestingly, many peptides were identified distributed throughout the polyprotein and were distinct of the known predicted cleavage sites, potentially indicating stable degradation fragments and/or potential cleavages from an undetermined protease (Fig. 2B). Alignment of the polyprotein sequences from related enterovirus B viruses revealed that many of these potential novel cleavage sites are conserved such as within VP1, VP3, 2C, 3C, and 3D (Fig. S1). Moreover, mapping the potential cleavage sites onto known structures of CVB3 (VP1, VP2, VP3 and VP4) 3C and 3D polymerase showed that some target cleavage sites are exposed and may be accessible to proteases (Fig. S2). In summary, identification of peptides associated with known CVB3 cleavage sites within the polyprotein validates the TAILS approach in virus-infected cells.

### TAILS analysis of host substrates during CVB3 infection

**Fig 3:**
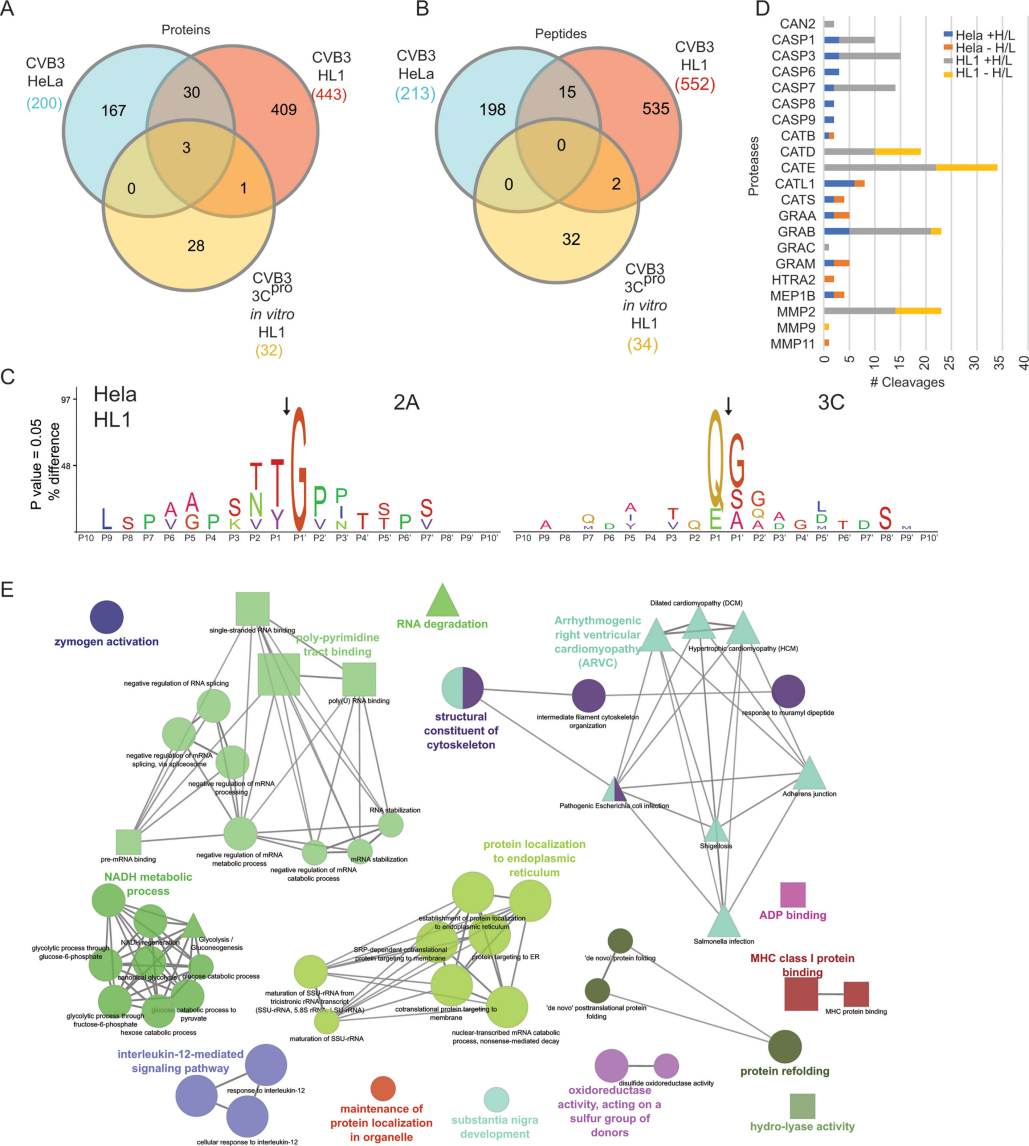
Identification of host cellular proteome in CVB3-infected cells. Venn diagrams of high-confidence proteins (**A**) and peptides (**B**) identified in TAILS analysis of CVB3-infected HeLa and HL-1 cells with comparisons with *in vitro* TAILS analysis of CVB3 3C^pro^ in HL-1 cell lysates (32). High-confidence substrates that had a positive H/L ratio outside the linear range were analyzed. (**C**) Ice logo of amino acid adjacent to the cleavage sites of candidate 2A^pro^ and 3C^pro^ substrates from TAILS HeLa and HL-1 data sets. (**D**) Bar graph of cellular protease cleavage sites using TopFinder inferred by TAILS high candidate substrates in CVB3-infected cells. Both positive and negative H/L ratio substrates were graphed. Calpain-2 (CAN2), caspase 1 (CASP1), caspase 3 (CASP3), caspase 6 (CASP6), caspase 7 (CASP7), caspase 8 (CASP8), caspase 9 (CASP9), cathepsin B (CATB), cathepsin (CATD), cathepsin E (CATE), procathepsin L (CATL1), cathepsin S (CATS), granzyme A (GRAA), granzyme B (GRAB), granzyme C (GRAC), granzyme M (GRAM), serine protease HTRA2, mitochondrial (HTRA2), meprin A subunit B (MEP1B), collagenase type IV (MMP2), matrix metalloproteinase-9 (MMP9), and stromelysin-3 (MMP11). (**E**) Network analysis of TAILS high-confidence proteins in CVB3-infected HeLa and HL-1 cells. Databases used for pathway and GO term analyses are depicted by node shapes with GO term biological processes (ellipse), GO term molecular function (rectangle), and Kyoto Genes and Genomes (KEGG) pathways (triangle).

We next examined host protein substrates identified by TAILS in CVB3-infected cells. For CVB3-infected HeLa and HL-1 samples, 453 and 694 substrates, (high to low H/L ratio) consisting of 508 and 860 peptides, respectively, were identified (Fig. S3 and S4; Data S1). Two hundred (HeLa) and 443 (HL-1) of the protein substrates had positive H/L ratios and were considered high-confidence candidates and of which 33 were found in both the HeLa and HL-1 data sets (Fig. 3A). Two hundred thirteen and 552 cleaved neo-N-terminal peptides were identified, identifying cut sites in individual proteins in this data set for the HeLa and HL-1 substrates, respectively (Fig. 3B). Previously identified enterovirus protease substrates were detected in this data set including DDX58 (HeLa), MAVS (HL-1), RELA (HL-1), HNRNPD (HeLa) HNRNPM (HeLa/HL-1), HNRNPK (HeLa/HL-1), PTBP1 (HeLa/HL-1), YAP1 (HeLa), PABP3 (HeLa), LIG3 (HeLa), CBX8 (HeLa), GTPBP3 (HeLa), HLTF (HeLa), CCT2 (HeLa), SF3B2 (HeLa), KRT18 (HeLa), PUF60 (HeLa), NEDD1 (HeLa), ARHGAP12 (HeLa), LSM14A (HeLa), Herc1, RBM4b, Scrib, and Arpc5 (HL-1) (23, 24, 31, 32, 53–55). The HNRNPM and PTBP1 peptides (GGGGAGGSVPGIER; AAGLSVPNVHGALAPLAIPSAAAAAAASR) identified in both the CVB3-infected HeLa and HL-1 TAILS data sets reveal that the cleavage site is conserved in both species. We also compared the CVB3-infected HeLa and HL-1 data sets to the *in vitro* cleavage TAILS substrates using CVB3 3C^pro^ (32) (Fig. 3A and B). Comparing these three datasets, few substrates overlapped. Three substrates, HNRNPK, HNRNPM, and Actin, were identified between all three data sets (Fig. 3A; Data S1). An additional 275 (HeLa) and 290 (HL-1) substrates and 299 (HeLa) and 311 (HL-1) peptides were identified where the natural or neo-N-terminus had a reduced H/L ratio in infected cells indicating loss of the protein substrate after initial cleavage (Fig. S4; Data S1).

Inference of proteases responsible for host protein cleavage was determined by investigating the adjacent residues of the TAILS-identified cleavage site. The enterovirus 3C^pro^ cleaves preferentially between Gln↓Gly, Gln↓Asn, Gln↓Ser, and Gln↓Ala and has a weaker preference for a P1 Glu, while the 2A^pro^ has more flexibility in the P1 site (Tyr, Thr, Ala, Val, Phe, Arg, or Leu) and preference for a P2 thr, Asn, Val, Gly, or Ser but almost exclusively requires a Gly in the P1’ site (17, 31, 32). By these criteria, predicted TAILS substrates with 3C^pro^ or 2A^pro^ cleavage sites were aligned and an Ice logo was generated (Fig. 3C). 3C^pro^ and 2A^pro^ substrates represented ~10% of all TAILS substrates identified. In addition to viral cleavage sites, corroborated high-confidence TAILS-generated cleavage sites by 21 cellular processes in CVB3-infected cells were identified using TopFinder (Fig. 3D) (45). Some were specific to the cell line, for example, predicted cleavages by cathepsin D, E, and MMP2 were identified exclusively in the CVB3-infected HL-1 data set with some of the highest number of events of all the identified proteases, while substrates of caspases 6, 8, 9, cathepsin B, S, procathepsin L, granzyme M, serine protease HTRA2, and Meprin A were identified exclusively with the CVB3-infected HeLa data set.

Network KEGG pathway analysis of high H/L ratio protein substrates detected in both the CVB3-infected HeLa and HL1 data sets showed conservation across a variety of pathways (Fig. 3E; Fig. S5 and S6). As expected, genes involved in RNA metabolism such as RNA stability and splicing were enriched. Interestingly, proteins that are enriched include those that function in protein folding and protein localization to the endoplasmic reticulum. In summary, TAILS points to several cellular pathways that are modulated, some of which are likely to promote infection.

### Analysis of host proteins in CVB3- and poliovirus-infected cells

**Fig 4:**
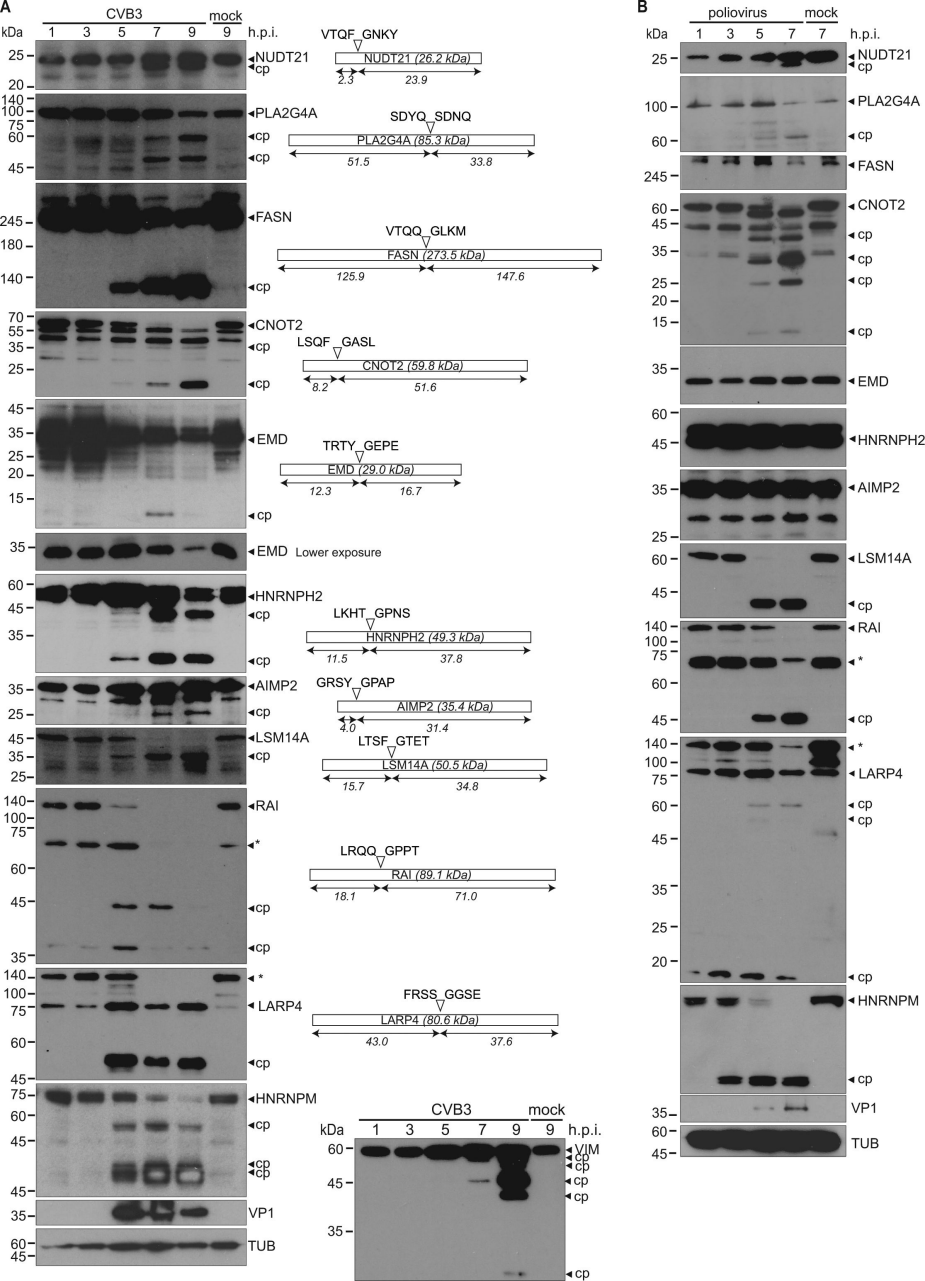
Validation of cleavage of candidate TAILS protein targets in CVB3-infected cells. Immunoblotting of indicated proteins in (**A**) CVB3-infected HeLa cells (MOI 10) and (**B**) poliovirus-infected HeLa cells (MOI 10). Shown in (A) (right) are the schematic of proteins, the predicted mass of cleavage fragments, and the predicted cleavage site with P4-P1 and P1′-P4’ amino acids based on the TAILS detected peptide. cp, cleavage product; *, non-specific, unclassified product. Representative immunoblots from at least two independent experiments.

To prioritize TAILS substrates for validation and functional studies, we focused on candidates that had a high H/L ratio outside the linear range, predicted to be CVB3 3C^pro^ or 2A^pro^ substrates, and cross-referenced to previous reports on functional studies with enterovirus infection (i.e. genetic knockdown/knockout screens) and interactomes associated with the viral RNA genome (Data S1). We monitored select candidate proteins in CVB3-infected HeLa cells by immunoblotting analysis (Fig. 4A). From the candidate targets, we detected either loss of full-length protein over the course of CVB3 infection and/or the presence of cleavage products. Substrates predicted to be cleaved by the 3C^pro^ include PLA2G4A, FASN, and RAI (PPP1R13L), while 2A^pro^ predicted targets include NUDT21, CNOT2, EMD, HNRNPH2, AIMP2, LSM14A, and LARP4. Similar to HNRNPM (40), RAI, LSM14A, LARP4, and CNOT2 showed complete loss of full-length protein during the course of infection. Most candidate proteins such as PLA2G4A, FASN, HNRNPH2, and LARP4 showed detectable cleavage products at 5 h.p.i. that were stable at later times of infection. Moreover, the majority of candidate substrates resulted in cleavage fragments that were predicted by the TAILS analysis (Fig. 4A). For instance, HNRNPH2 cleavage led to two cleavage fragments of ~11 and 38 kDa, which are the predicted cleavage fragments inferred by TAILS analysis, targeting cleavage at LLHT↓GPNS (Fig. 4A). For some substrates such as PLA2G4A, RAI, and VIM, TAILS analysis predicted a single cleavage site, yet multiple cleavage products were detected indicating that the proteins are targeted at other sites by viral or cellular proteases (Fig. 4A). This was not unexpected as some peptides may not be detected by mass spectrometry due to degradation of protein fragments in the proteasome or by caspases—additionally, TAILS analysis looked at a single time point within the replication cycle so temporal changes may not have been detected.

To determine whether cleavage of candidate proteins occurs in another enterovirus infection, PV-infected cell lysates were analyzed by immunoblots (Fig. 4B). As observed in CVB3-infected cells, CNOT2, PLA2G4A, NUDT21, LSM14A, RAI, and LARP4 were cleaved under PV infection. By contrast, EMD and AIMP2 did not show cleavage, suggesting specificity of target proteins under distinct enterovirus infections. These results demonstrated that several host proteins are targeted in at least two enterovirus infections, possibly pointing to a common strategy among enteroviruses.

Finally, we monitored cleavage of select candidates (where antibodies cross-reacted) in CVB3-infected mouse HL-1 cells (Fig. S7). Similar to that observed in CVB3-infected HeLa cells, full-length FASN protein levels decreased, and a stable product was detected as infection progresses. Full-length RAI but not EMD protein levels decreased during infection. These results indicated that there is specificity in host cell substrate targeting and cleavage in different enterovirus infection cell types.

### Host proteins cleaved by viral proteases *in vitro*

**Fig 5:**
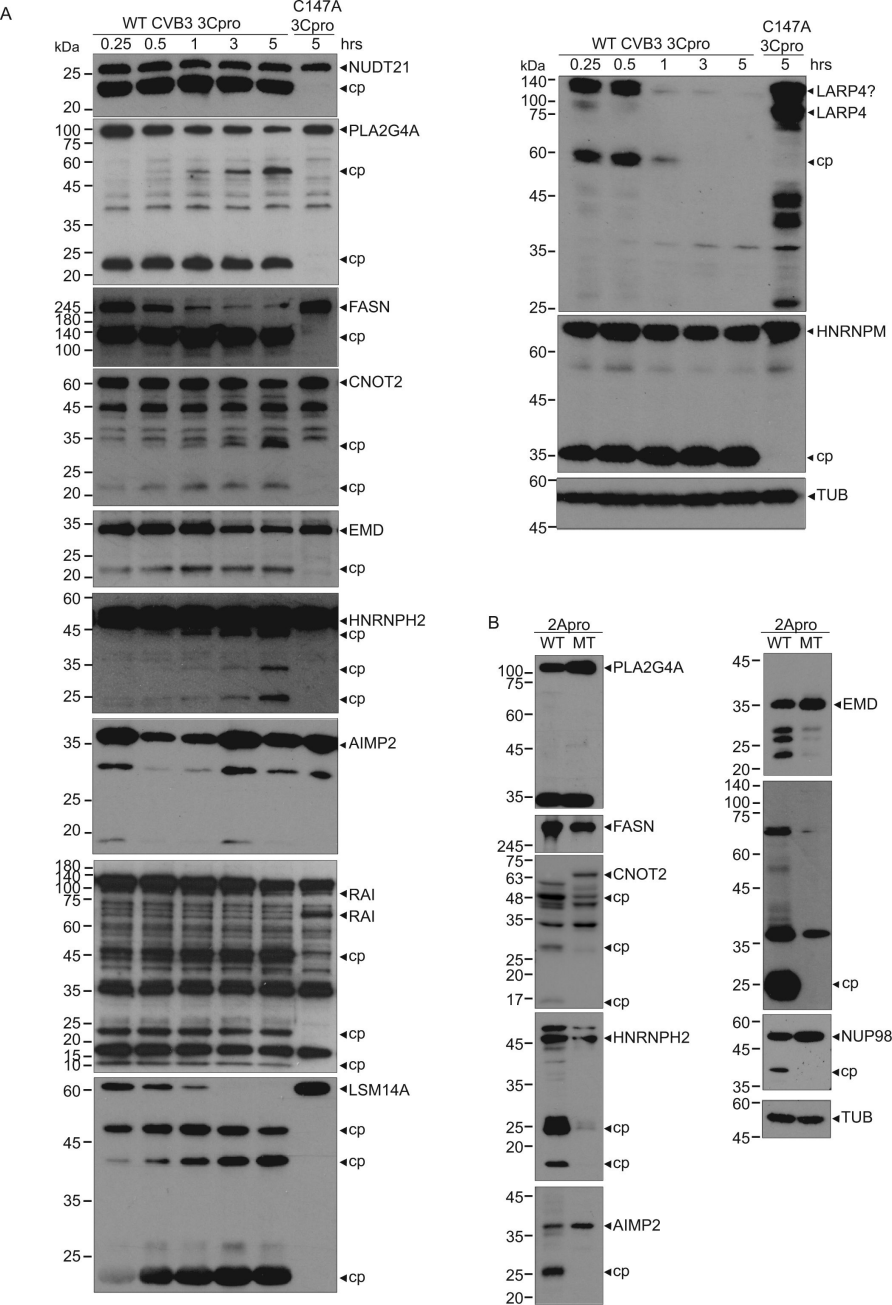
Validation of candidate of TAILS protein targets using *In vitro* cleavage assays. Immunoblots of indicated proteins in HeLa cell lysates incubated with purified recombinant (**A**) wild-type or catalytically inactive (C174A) CVB3 3C protease or (**B**) wild-type or catalytically inactive CVB3 2A protease (C57A). cp, cleavage product. Representative immunoblots from at least two independent experiments.

To examine whether candidate substrates are direct targets of CVB3 3C^pro^ or 2A^pro^, we performed *in vitro* cleavage assays by incubating purified recombinant wild-type or catalytically inactive 3C^pro^ or 2A^pro^ in HeLa cell lysates. We focused on substrates that were predicted to be cleaved by 3C^pro^ or 2A^pro^. Addition of wild-type but not catalytically inactive mutant (C147A) 3C^pro^ to cell lysates resulted in detection of cleavage fragments of PLA2G4A, FASN, RAI, HNRNPH2, CNOT2, NUDT21, LARP4, LSM14A, and EMD, indicating that these substrates are direct targets of 3C^pro^ (Fig. 5A). Of these, only PLA2G4A, RAI, and FASN resulted in detectable cleavage products of the same mass as predicted from the TAILS analysis. AIMP2, EMD, HNRNPH2, NUDT21, LSM14A, LARP4, and CNOT2 are predicted to be targets of 2A^pro^. *In vitro* cleavage assays using purified wild-type 2A^pro^ resulted in detectable cleavage products of AIMP2, EMD, HNRNPH2, and CNOT2 (Fig. 5B). Cleavage was specific as incubation of catalytically inactive 2A^pro^ (C57A) did not alter full-length protein levels nor were cleavage fragments detected. FASN and PLA2G4A, substrates of 3C^pro^, were not cleaved in assays containing 2A^pro^. In summary, we demonstrated *bona fide* direct cellular substrates of 3C^pro^ and 2A^pro^.

### Functional relevance of target proteins for CVB3 infection

**Fig 6:**
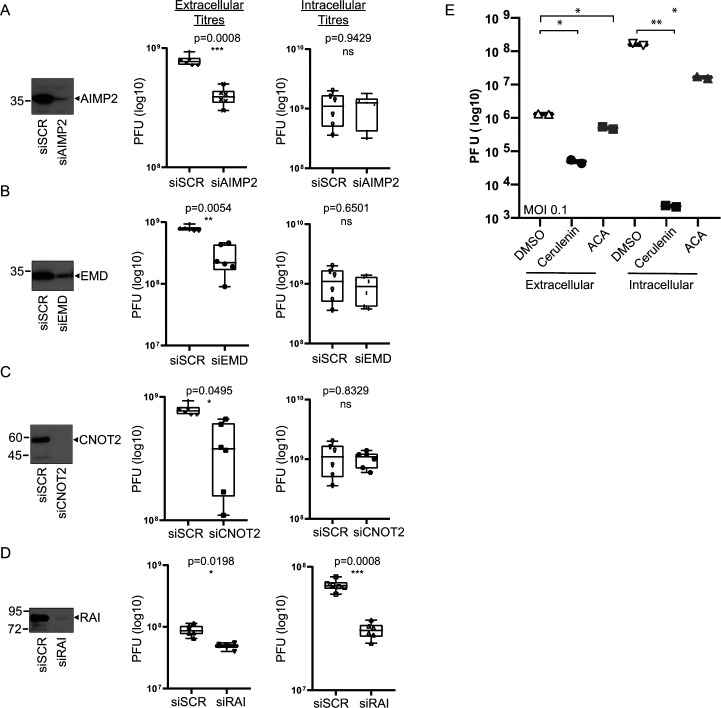
Depletion of TAILS candidate proteins in CVB3-infected cells. Immunoblots of HeLa cells transfected with control siRNA (siSCR) or siRNAs directed against (**A**) AIMP2, (**B**) EMD, (**C**) CNOT2, or (**D**) RAI. siRNA-treated cells (48 h) were infected with CVB3 with an MOI 1.0 (**A, B, C**) or 0.1 (**D**) for 16 h. Extracellular and intracellular viral yields were detected by plaque assay. Shown are box plots of log_10_ PFU with means and 95% confidence intervals from three independent experiments with at least two technical replicates. (**E**) Extracellular and intracellular viral titers collected from CVB3-infected (MOI 0.1) HeLa cells were pre-treated with cerulenin (45 M) and ACA (20 M). *P*-values from nested one-way *t*-test are indicated.

To determine the functional relevance of the targeted substrates for infection, we first depleted select target proteins by siRNA, followed by infection with CVB3 (MOI 1.0) and determined viral titers at 16 h post infection (Fig. 6). siRNA depletion resulted in >90% knockdown of AIMP2, EMD, CNOT2, and RAI protein expression (Fig. 6A through D, left). CVB3 infection (MOI 1.0) of AIMP2-, EMD-, and CNOT2-depleted cells resulted in a statistically significant decrease in extracellular viral titer (Fig. 6A through C). CVB3 infection (MOI 0.1) of RAI-depleted cells led to a statistically significant decrease in both extracellular and intracellular viral titers (Fig. 6D).

Modulation of lipid metabolism is key to promoting enterovirus infection (56, 57). Of note, candidate proteins involved in this pathway include FASN and cytosolic phospholipase, PLA2G4A [(58) 2008, Group IVA phospholipase A2 is necessary for the biogenesis of lipid droplets]. To examine their roles in infection, we monitored the viral yield of CVB3-infected cells treated with inhibitors that block FASN and PLA2G4A enzymatic activities. CVB3-infected cells with the FASN inhibitor, cerulenin, resulted in a decrease in CVB3 viral yield, supporting previous reports that FASN is required for virus infection (Fig. 6E) (59). Similarly, CVB3-infected cells treated with the PLA2GF4A inhibitor, ACA, also reduced intracellular and extracellular CVB3 viral yield (Fig. 6E). In summary, these results demonstrated that a subset of TAILS-identified target proteins are necessary for productive virus infection.

### Role of EMD in CVB3-infected cells

**Fig 7:**
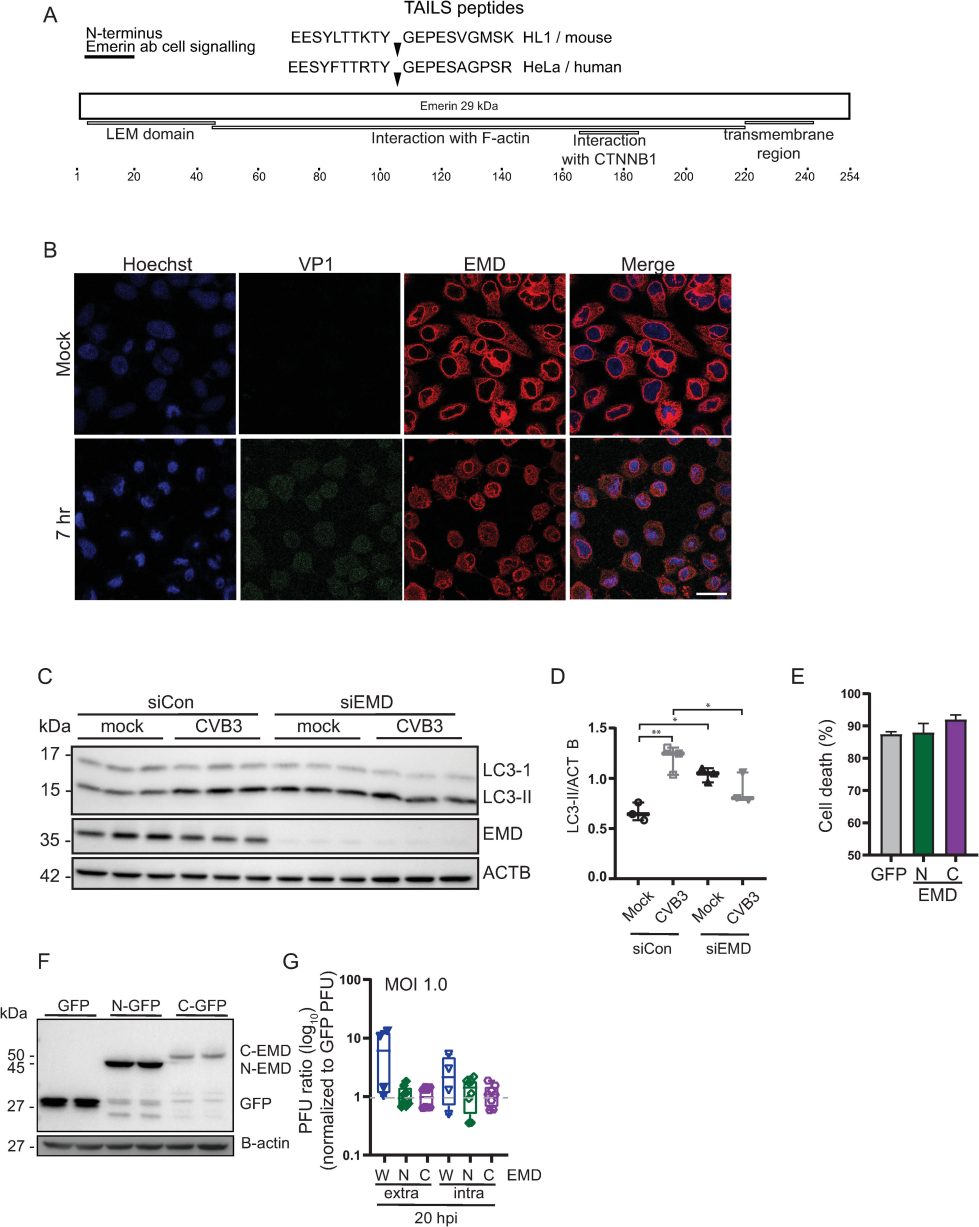
EMD promotes autophagy and CVB3 infection. (**A**) Schematic of EMD with annotated domains and the TAILS peptides identified in CVB3-infected HeLa and HL-1 cells. (**B**) Immunofluorescence of EMD in mock- or CVB3-infected HeLa cells (MOI 10). Scale bar 20 µm. (**C**) Immunoblots of indicated proteins in control siRNA or EMD siRNA-treated mock- or CVB3-infected cells. (**D**) Quantitation of (**C**) LC3-II immunoblots. (**E**) Immunoblots of GFP-N-EMD and GFP-C-EMD from HeLa cells transfected with expression plasmids containing N- and C-terminal EMD cleavage fragments. (**F**) Viability of cells transfected with GFP-tagged N-terminal or C-terminal plasmids (24 h post transfection) by trypan blue dye exclusion. (**G**) Extracellular and intracellular virus titers from CVB3-infected (MOI 1.0) HeLa cells transfected with GFP-N-EMD and GFP-C-EMD, collected at 20 h post infection. Shown are box plots of plaque assay (PFU) from at least three independent experiments of CVB3-infected cells transfected with the indicated plasmids normalized to that of CVB3-infected cells transfected with GFP-expressing plasmid.

We next examined the functional role of specific candidate proteins in CVB3 infection. EMD is a ubiquitous 29 kDa integral membrane protein that localizes to the nuclear membrane and has several diverse cellular functions, including chromatin tethering, gene regulation, mitosis, and autophagy (60, 61). EMD possesses a C-terminal transmembrane region, an N-terminal LAP2, emerin, MAN1 (LEM) protein-interacting domain, an F-actin interacting domain, and a β-catenin one interacting domain (Fig. 7A). EMD is cleaved within the F-actin interacting domain in both CVB3-infected HeLa and HL-1 cells, which releases the N-terminal LEM domain (Fig. 7A). We first analyzed the subcellular localization of EMD in mock- vs CVB3-infected cells by immunofluorescence. In mock-infected cells, EMD antibody staining was detected in the cytoplasm with a strong nuclear peripheral enrichment (60). In CVB3-infected cells, EMD staining using an antibody raised to the N-terminal domain was more diffuse throughout the cytoplasm and less enriched at the nuclear periphery (Fig. 7B), suggesting that cleavage altered the subcellular localization of EMD.

Given that EMD is linked to autophagy and that enterovirus infection leads to modulation of this pathway (61–64), we examined whether depletion of EMD by RNAi has an effect on autophagy in CVB3-infected cells by monitoring the levels of lipidated LC3 (LC3-II). Knockdown of EMD in mock-infected cells resulted in an increase in LC3-II compared to that in scrambled siRNA-treated cells indicating an increase in autophagosome formation, suggesting that EMD dampens basal autophagy (Fig. 7C). Under CVB3 infection, LC3-II levels are increased in line with previous reports that CVB3 infection promotes a noncanonical autophagic pathway (65). By contrast, CVB3 infection of EMD-depleted cells resulted in a reproducible, albeit minor, decrease in virus-induced LC3 lipidation, consistent with the role of EMD protein in promoting autophagy (Fig. 7D). Collectively, our results showed that EMD contributes to CVB3 infection in part by promoting and subverting the autophagic pathway.

Cleavage of EMD during CVB3 infection likely blocks its cellular functions; however, it is also possible that the cleaved N-terminal or C-terminal fragments have a role in infection. As shown in Fig. 5, the cleaved EMD N-terminal fragment is detected and moderately stable during infection. To examine this, we generated expression plasmids that contain GFP-tagged full-length wild-type, N-terminal, or C-terminal fragments of EMD based on the TAILS-identified cleavage site. Transfection of the full-length wild-type, N-terminal, or C-terminal EMD fragment constructs was not toxic and did not affect cell viability (Fig. 7E). Moreover, immunoblotting showed stable N- and C-terminal EMD proteins (Fig. 7F). We transfected HeLa cells with the full-length, N-terminal, or C-terminal EMD-GFP constructs followed by CVB3 infection, and monitored viral yield (Fig. 7G). In this experiment, viral yield was also monitored in cells transfected with the GFP tag alone, which is used for normalization of the effects of cells expressing the full-length, N-terminal, or C-terminal EMD-GFP proteins. We calculated the ratio fold plaque forming units of cell expressing full-length, N-terminal, or C-terminal EMD-GFP proteins over that of GFP-expressing cells. Overexpression of the full-length EMD-GFP resulted in an ~8-fold increase in CVB3 extracellular viral yield as compared to that in GFP-expressing cells (Fig. 7G). This result is in line with depletion studies (Fig. 6B) indicating that EMD expression promotes CVB3 infection. By contrast, expression of the N-terminal or C-terminal fragments did not have an effect on viral yield compared to GFP-expressing cells (Fig. 7G). These results demonstrated that EMD promotes CVB3 extracellular viral yield and suggested that cleavage of EMD reduces viral yield.

### AIMP2 contributes to the CVB3 life cycle

**Fig 8:**
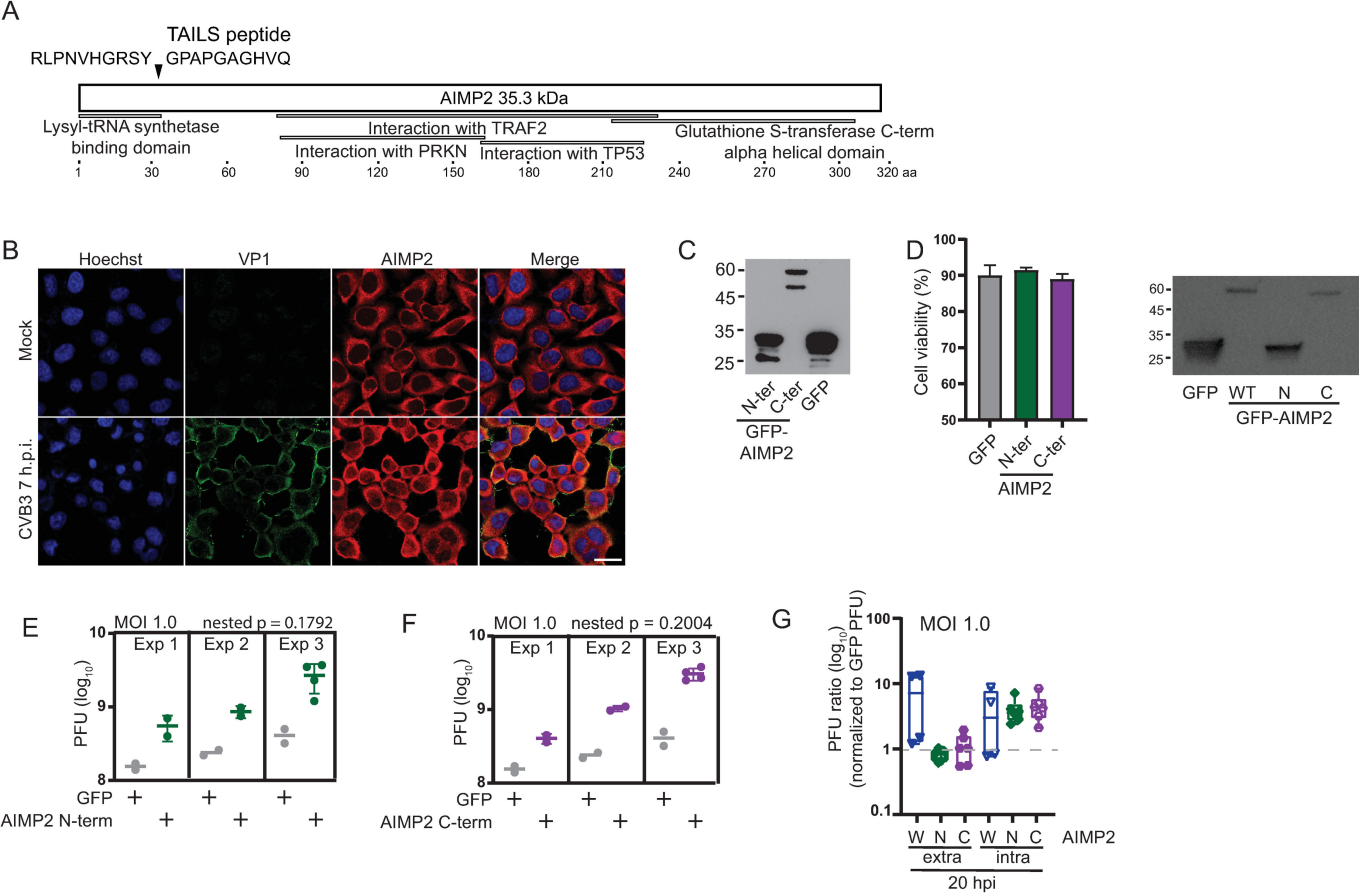
Cleavage fragments of AIMP2 promotes CVB3 infection. (**A**) Schematic of AIMP2 with annotated binding domains and the TAILS peptides identified in CVB3-infected HeLa and HL-1 cells. (**B**) Immunofluorescence of AIMP2 in mock- or CVB3-infected HeLa cells (MOI 10). Scale bar 20 µm. (**C**) Immunoblots of HeLa cells transfected with expression plasmids containing GFP-tagged N-terminal and C-terminal AIMP2 cleavage fragments. (**D**) Viability of cells transfected with GFP-tagged N-terminal or C-terminal AIMP2 plasmids by trypan blue dye exclusion assay. Viral yield measured by plaque assay (PFU) of CVB3-infected cells transfected with GFP control plasmid or GFP-tagged (**E**) N-terminal or (**F**) C-terminal AIMP2 cleavage fragment plasmids. Shown are three independent experiments, displaying the averages of at least three technical measurements. (**G**) Quantitation of (**E**) and (**F**) by box plots and normalizing PFU of HeLa cells transfected N-terminal and C-terminal AIMP2 cleavage fragment plasmids normalized to that of cells transfected with GFP.

We next examined the role of another host protein candidate, the 35 kDa AIMP2, which is a tumor suppressor protein that is part of a multi-tRNA synthetase complex and is proposed to act as a scaffold as well as a regulator of signaling pathways such as the NFκB pathway and apoptosis (66, 67). Based on TAILS, AIMP2 is cleaved near the N-terminus, resulting in the separation of the lysyl-tRNA synthetase binding domain from the rest of the protein, which contains a glutathione S-transferase alpha helical domain as well as region known to interact with Parkin RBR E3 ubiquitin protein ligase (PRKN), TNF receptor-associated factor 2 (TRAF2), and tumor protein p53 (Fig. 8A).

We first investigated the subcellular localization of AIMP2 under CVB3 infection by immunofluorescence. Under basal conditions, AIMP2 is mainly detected in the cytoplasm, whereas under CVB3 infection, AIMP2 signal is altered with relatively more signal in the nucleus. AIMP2 is cleaved under CVB3 infection, leading to stable expression of the cleaved C-terminal fragment (Fig. 4A). To determine whether the cleaved fragments have a role in infection, we expressed GFP-tagged N-terminal or C-terminal AIMP2 fragments in HeLa cells infected with CVB3. Expression of GFP-tagged N-terminal and C-terminal AIMP2 in HeLa cells did not significantly affect cell viability compared to GFP alone-expressing cells (Fig. 8C and D). Compared to GFP-expressing cells, cells expressing GFP-N-AIMP2 or GFP-C-AIMP2 resulted in reproducible increase in intracellular CVB3 yield by approximately ~3- to 5-fold at 20 h.p.i. These results showed that expression of the cleaved fragments of N-term or C-term fragments of AIMP2 facilitates CVB3 infection.

### Modulation of NFĸB pathway by AIMP2

We next examined the mechanism by which cleaved AIMP2 affects CVB3 infection. AIMP2 has been shown to regulate TNFα-induced activation of NFκB (66, 67). NFĸB is sequestered by the nuclear factor of kappa light polypeptide gene enhancer in B-cells inhibitor alpha (Iκκα)-containing complex and upon activation, NFĸB translocates to the nucleus to induce transcription of pro-inflammatory genes. The NFĸB pathway regulates a number of genes involved in inflammation, immunity, cell proliferation, differentiation, and survival (68).

**Fig 9:**
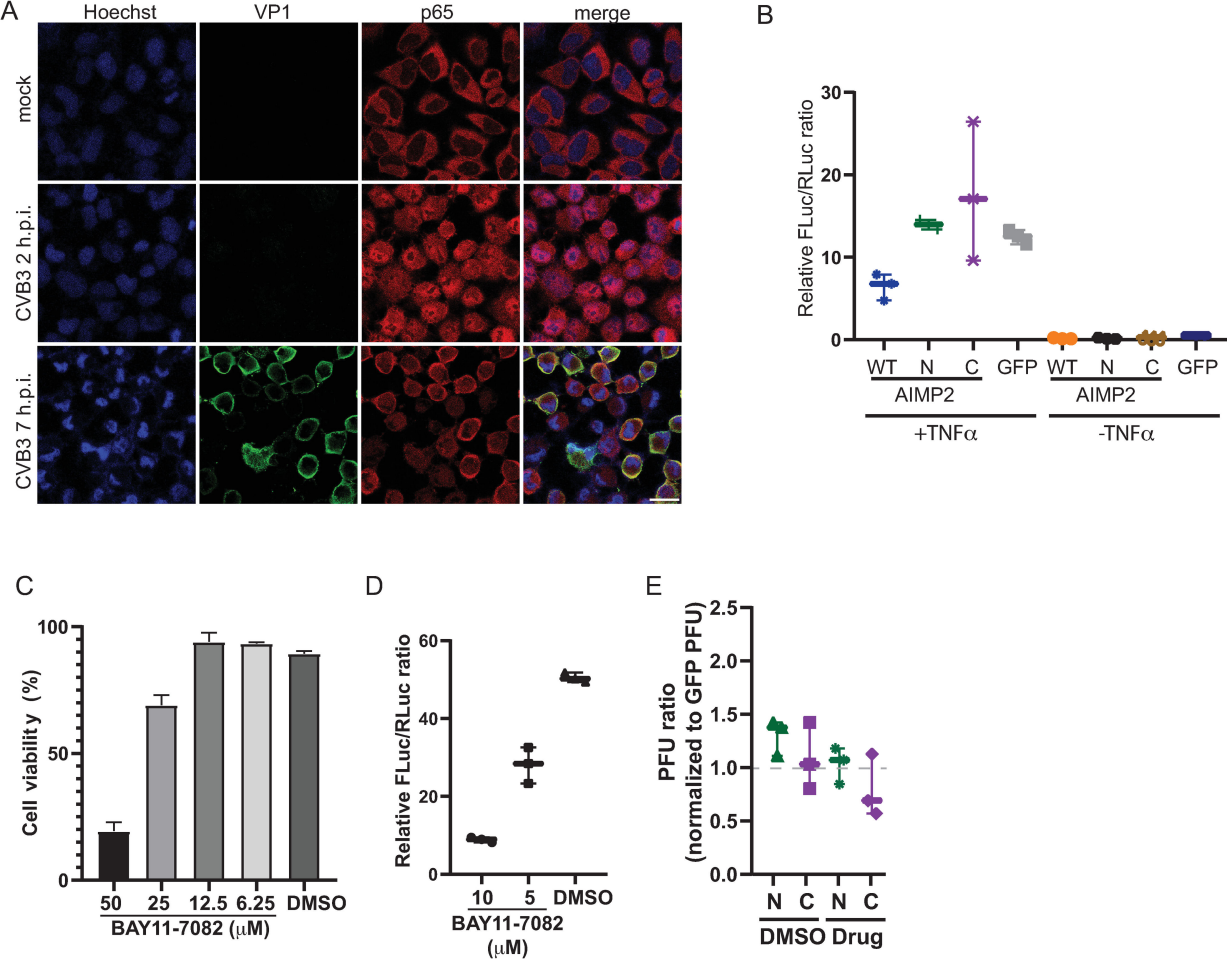
Cleavage fragments of AIMP2 modulate NFκB signaling. (**A**) Immunofluorescence of p65 in mock- and CVB3-infected HeLa cells. Scale bar 20 µm. (**B**) Induction of NFκB-responsive firefly luciferase reporter in TNFα-treated HeLa cells transfected with plasmids expressing GFP-tagged wild-type AIMP2, N-terminal or C-terminal cleavage fragments of AIMP2. Cells were co-transfected with a Renilla luciferase reporter for normalization. Shown are the ratio of firefly luciferase activities normalized to GFP-expressing cells. (**C**) Viability of cells treated with indicated drug concentrations. (**D**) Normalized NFκB-responsive firefly luciferase reporter in TNFα-treated HeLa cells with the indicated concentrations of BAY11-7082 for 20 min. (**E**) Relative viral titers of HeLa cells transfected with either N-terminal or C-terminal AIMP2 and treated with BAY11-7082 (10 µm) 30 min prior to infection (MOI 0.1) for 16 h. The values are normalized with GFP-transfected infected cells and presented as averages ± SD from at least three independent experiments.

We first monitored NFĸB subcellular localization in CVB3-infected cells by immunofluoresence using an antibody to p65, which is a component of the NFĸB transcription factor complex. As early as 2 h.p.i. CVB3 infection p65 re-localized from the cytoplasm to the nucleus, indicating activation of the NFĸB pathway (Fig. 9A). At later times of infection, at 7 h.p.i., the majority of the p65 signal is redistributed to the cytoplasm. Thus, CVB3 infection modulates NFĸB re-localization in a temporal manner and in line with previous reports that CVB3 infection modulates the NFĸB pathway to promote infection (69).

We investigated whether the cleaved N-terminal or C-terminal fragments of AIMP2 modulates the NFĸB pathway. Toward this, we expressed full-length, N-terminal, or C-terminal AIMP2 and monitored NFĸB activation in HeLa cells induced by TNFα treatment. NFκB activation was monitored by co-transfecting constructs containing a NFĸB-responsive firefly luciferase reporter and a control Renilla luciferase reporter for normalization of transfection efficiency. Treating HeLa cells with TNFα for 2 h resulted in an increase in firefly luciferase activity indicative of NFĸB-mediated transcriptional activation (Fig. 9B). Transfection of GFP-AIMP2 (full-length) resulted in a decrease in NFĸB-firefly luciferase (FLuc) activity compared to that transfected with the GFP only control plasmid, in line with a previous report that AIMP2 inhibits the NFκB pathway (Fig. 9B) (67). By contrast, expression of the GFP-N-AIMP2 or GFP-C-AIMP2 restored the NFĸB-Fluc activity to that of the GFP-transfected control cells, indicating that the AIMP2 fragments are unable to dampen the NFĸB pathway.

To determine whether the increase in CVB3 viral yield in cells expressing N-terminal or C-terminal AIMP2 fragment is due to the effects on the NFκB pathway, we treated cells with the NFĸB pathway inhibitor, BAY11-7082, which inhibits the phosphorylation of IκB-α (70). First, we examined the effects of the drug on NFκB activation and cell viability. Treating cells with TNFα and increasing concentrations of BAY11-7082 (5 and 10 µM) resulted in a dose-dependent decrease in NFĸB-FLuc activity, which, at these inhibitor concentrations, were not toxic to cells (Fig. 9C and D). Higher concentrations (25 and 50 µM) of BAY11-7082 were more toxic to cells (Fig. 9C). We next examined effects of the NFĸB inhibitor on CVB3-infected cells transfected with either the N-terminal or C-terminal AIMP2. Compared to untreated cells, CVB3-infected cells treated with the NFκB inhibitor resulted in a decrease in viral yield (Fig. 9E). These results suggested that reduced NFĸB signaling via expression of the N-terminal or C-terminal AIMP2 contributes to CVB3 infection.

## DISCUSSION

Targeting host proteins by viral proteases is a general viral strategy used by most, if not all, viruses to promote infection. Viral proteases target host proteins to modulate a number of cellular processes, either to redirect and usurp cellular factors to promote specific steps of virus infection or to evade antiviral immune responses, thus identification of targeted host proteins is key to fully identifying the cellular networks modulated by virus infection. Advances in N-terminomic approaches have been instrumental in identifying the host proteins that are cleaved by viral proteases *in vitro* or in virus-infected cells (36, 71, 72). Here, we used for the first time the TAILS N-terminomics approach to identify the network of host protein substrates in CVB3-infected cells. We validated that a subset of host proteins are cleaved in CVB3-infected cells and are direct targets of CVB3 3C^pro^ using an *in vitro* cleavage assay. Functional studies using knockdown and overexpression studies showed that 3C^pro^-mediated cleavage fragments of EMD and AIMP2 modulates CVB3 infection. This study highlights the global repertoire of host protein substrates that are cleaved under CVB3 infection as a strategy to promote infection.

N-terminomics approaches have accelerated the identification of viral protease-specific targets and the degradome in virus-infected cells. N-terminomics analyses using TAILS and subtiligase-based approaches have been applied using *in vitro* cleavage assays with recombinant viral proteins revealing substrates of proteases of coronavirus, severe acute respiratory syndrome coronavirus 2 (SARS-CoV-2), flavivirus, Zika, enteroviruses, PV, and CVB3 (31–33, 35). Of note, Saeed et al. used the N-terminomics subtiligase-based positive selection approach to identify cleaved host proteins in CVB3-infected cells (31). In comparing the targets between the two N-terminomics approaches, there were a few target substrates that were identified by both approaches; however, in general, the majority of substrates identified in CVB3-infected cells were distinct, thus highlighting the utility of multiple approaches to identify the full repertoire of host cell protein targets (Fig. S2).

Based on the amino acid residues surrounding the predicted TAILS-identified cleavage site, we identified proteins that are targeted by CVB3 3C^pro^ and 2A^pro^, and for a subset, we validated cleavage by 3C^pro^ *in vitro* (Fig. 3 and 5). We also identified proteins that are predicted to be targeted by several cellular proteases (Fig. 3D), including caspases, metalloproteinases, granzymes, and cathepsins, suggesting that these enzymes are activated during infection. Activation of caspases is likely due to induction of apoptosis, which is associated late in enterovirus infections (73–75). It will be of interest to investigate the regulation of these other cellular proteases during CVB3 infection.

The network analysis of the proteins identified by TAILS revealed several cellular pathways targeted in CVB3-infected cells. The 3C^pro^-mediated cleavage of FASN and PLA2G4A (cytosolic phospholipase A2) in both PV- and CVB3-infected cells may suggest a general enterovirus strategy. FASN has been shown to contribute to lipid metabolism, which is important for replication for several plus-strand RNA viruses including CVB3, PV, and dengue virus (58, 76–78). In line with this, treating cells with the FASN inhibitor, cerulenin, resulted in a decrease in CVB3 yield (Fig. 6E). PLA2G4A specifically has not been linked directly to virus infection; however, cytosolic phospholipases have been associated with biogenesis and membrane remodeling of West Nile virus (47). Our studies showed that CVB3 infection is decreased when cells were incubated with ACA, a broad-spectrum phospholipase A(2) inhibitor (Fig. 6E). ACA likely targets other phospholipases, including adipocyte phospholipase A2 (PLA2G16), which has been reported to have a role in the delivery of enterovirus genomes to the cytosol prior to cellular detection of membrane permeation and subsequent viral clearance (79, 80). Despite being important for infection, cleavage of FASN and PLA2G4A hints that lipid synthesis and membrane remodeling are regulated during infection, possibly to facilitate specific steps of the viral life cycle. Alternatively, the N-terminal or C-terminal cleavage fragments may participate directly/indirectly in the viral life cycle.

We also identified EMD that is cleaved during CVB3 infection and is a substrate of 3C^pro^ (Fig. 4 and 5). Although EMD has been linked to virus budding and nucleocapsid maturation in herpesvirus infection (60, 81), to our knowledge, its role in RNA virus infection has not been reported. Our data suggest that CVB3 may require EMD to induce the lipidation stage of autophagy (Fig. 7C and D) and that depletion of EMD decreased extracellular CVB3 yield (Fig. 6B), suggesting a role of EMD in virus packaging and/or egress. EMD is directly linked to autophagy where phosphorylated EMD binds MAP1LC3B, leading to an increase in autophagosomes (61). Moreover, CVB3 infections subvert the autophagosome pathway and use microvesicles for virus particles to exit the host cell (82, 83). EMD is not cleaved to completion, possibly suggesting that its role in autophagy is tightly regulated during CVB3 infection. Further investigation of the timing and regulation of EMD in subverting the autophagosome pathways is warranted.

Several host proteins, such as RAI and AIMP2, associated with the NFκB pathway are cleaved during CVB3 infection (Fig. 4). The importance of NFκB in enterovirus lifecycles is complex as it is involved in evading antiviral responses and also in promoting viral infection (84, 85). The multifunctional AIMP2 is a non-enzymatic scaffold protein that facilitates the assembly of and is essential for the stability of the multi-aminoacyl-tRNA synthetase complex (multi-ARS); however, the functions of which are not fully understood (86, 87). Cleavage of AIMP2 by 3C^pro^ likely leads to disassembly of the multi-ARS complex, which may facilitate CVB3 infection. The multi-ARS has been linked to TNFα-mediated apoptosis through a decrease in NFĸB activity (67), where AIMP2 sequesters TRAF2 into a complex with c-IAP1, promoting the ubiquitination of TRAF2. Consistent with these findings, expression of AIMP2 led to decreased NFĸB reporter expression (Fig. 8); however, the expression of the cleaved fragments of AIMP2 did not, thus suggesting that cleavage of AIMP2 by viral 3C^pro^ disrupts this signaling pathway. It will be of interest to examine in more depth how the cleavage fragments inhibit NFκB pathway, possibly through modulation of the ubiquitination of TRAF2. Previous studies have shown that the splice variant of AIMP2 [AIMP2-DX2 (NM_001326607.1)] stabilizes TRAF2 by binding to TRAF2 but inhibiting the TRAF2-c-IAP1 interaction, thus inhibiting TRAF2 ubiquitination with normal NFĸB activity (88). The C-terminal AIMP2 cleavage product observed in our study is similar, but not identical, to the DX2 variant, so it is possible that this cleavage product results in a similar effect as observed with DX2. In addition to its role in NFĸB signaling, AIMP2 also has a pro-apoptotic function by interacting with p53 (89). Links between CVB3 infection, AIMP2 cleavage and apoptosis await further investigation.

In this study, we provide a comprehensive view of CVB3-driven degradome through TAILS N-terminomics, in both HeLa and the physiologically relevant HL1 cardiomyocyte cell lines. The identification of target host proteins provides a framework into the host protein networks that are targeted by the virus and induced by host responses, including some which may actively contribute to disease onset. Together with previous studies, this inventory of protein substrates expands our understanding of the complexity of the CVB3 lifecycle and highlights the importance of system-based approaches in the study of virus-host interactions.

## Data Availability

The MS proteomics data in this paper have been deposited in the ProteomeXchange Consortium (proteomecentral.proteomexchange.org) database under accession number PXD037570.

## SUPPLEMENTAL MATERIAL

Fig. S1 jvi.00498-24-s0001.pdf Comparison of cleavage sites from TAILS within an alignment of Enterovirus B polyprotein sequences.

Fig. S2 jvi.00498-24-s0002.pdf Locations of cleavages sites in select structures of Coxsackievirus B3 proteins.

Supplemental material jvi.00498-24-s0003.pdf Table S1; Fig. S3 to S7.

Supplemental data jvi.00498-24-s0004.xlsx Peptides.

ASM does not own the copyrights to Supplemental Material that may be linked to, or accessed through, an article. The authors have granted ASM a non-exclusive, world-wide license to publish the Supplemental Material files. Please contact the corresponding author directly for reuse.
