# Supplementary figures and images for: Identification of the proteolytic signature in CVB3-infected cells
Source: J Virol. 2024 Jul 2;98(7):e00498-24. doi: 10.1128/jvi.00498-24 (PMC11265341; doi:10.1128/jvi.00498-24)

VLOK Figure S

VP2

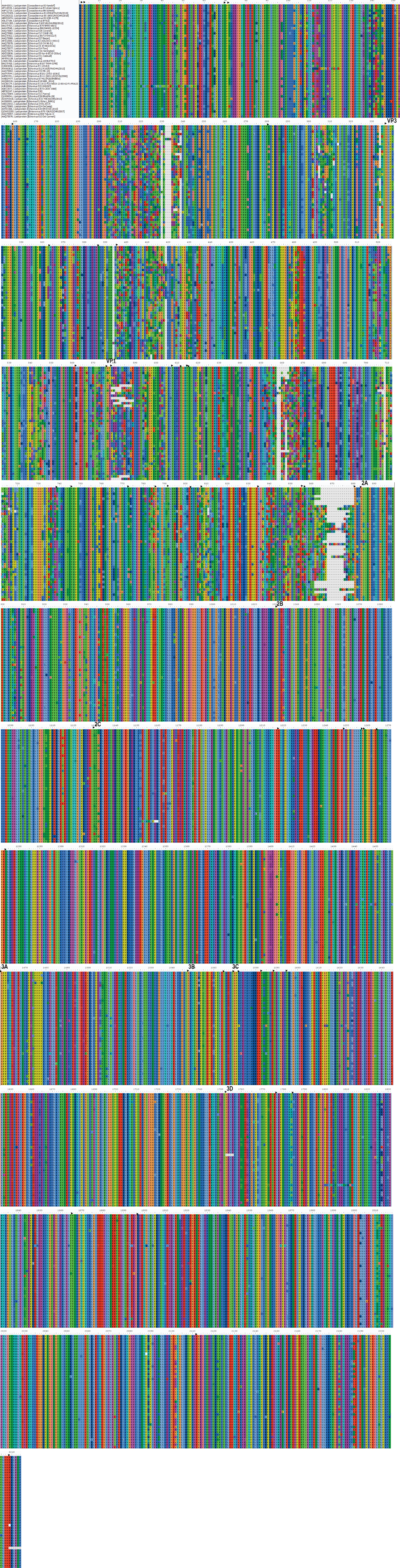

Supplement: Fig. S1 — Comparison of cleavage sites from TAILS within an alignment of Enterovirus B polyprotein sequences. [file jvi.00498-24-s0001.pdf]

A)

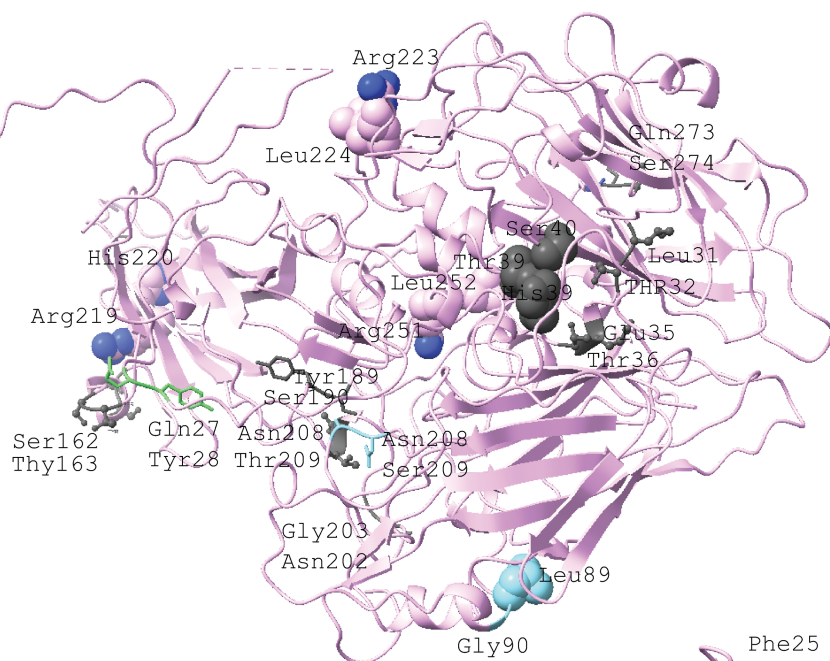

B)

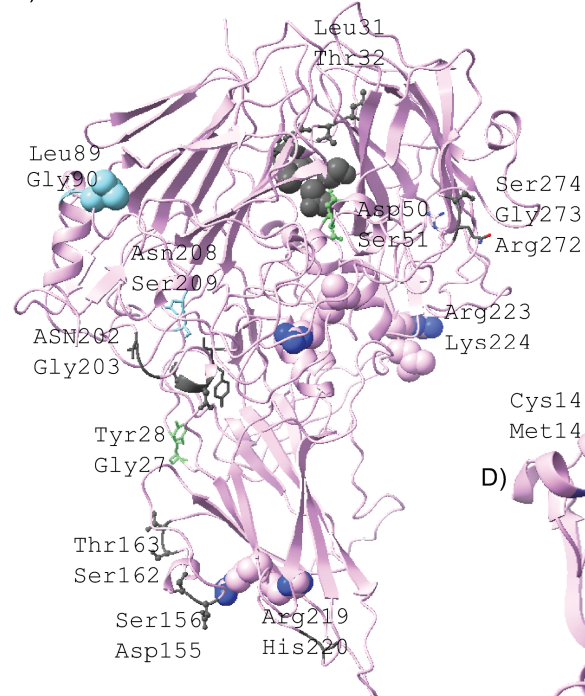

C)

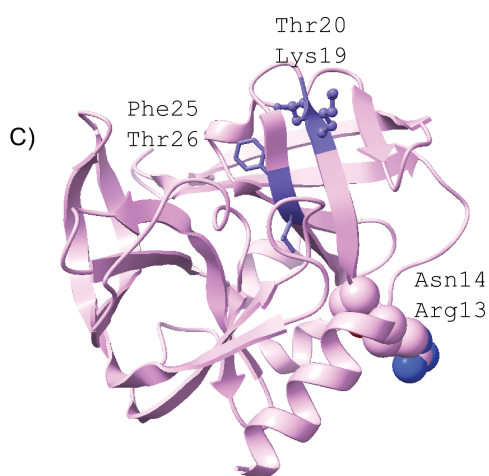

D)

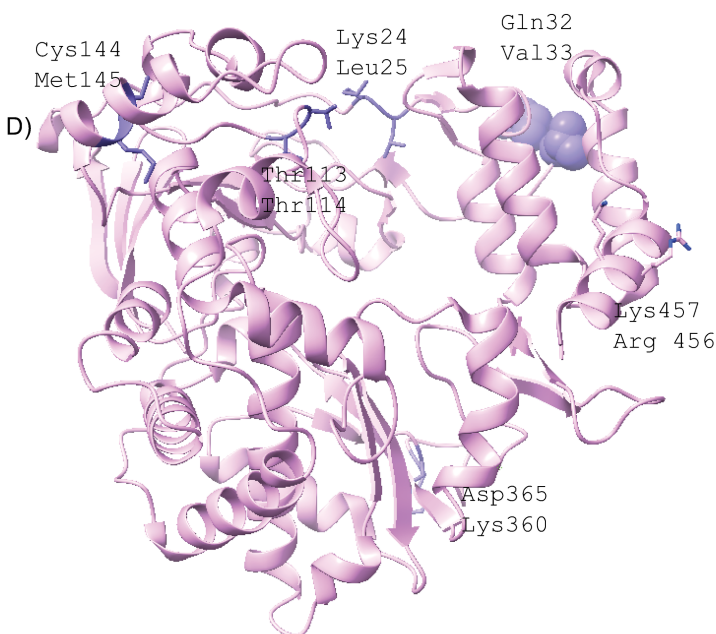

Supplement: Fig. S2 — Locations of cleavages sites in select structures of Coxsackievirus B3 proteins. [file jvi.00498-24-s0002.pdf]
