# Supplementary material for: Identification of the proteolytic signature in CVB3-infected cells
Source: J Virol. 2024 Jul 2;98(7):e00498-24. doi: 10.1128/jvi.00498-24 (PMC11265341; doi:10.1128/jvi.00498-24)

Supplementary Table 1: Peptide counts from CVB3-infected Hela and HL-1 samples. These counts represent the number of proteins and peptides detected after Mascot identification, which were used for quantification in Skyline as well as the number of proteins and peptides remaining post quantification and removal of repeated peptides (where the first appearance of a peptide was retained).

		Hela				HL1			
		TAILS		Pre-TAILS		TAILS		Pre-TAILS	
		Ndm	Nother	Ndm	Nother	Ndm	Nother	Ndm	Nother
Pre-Skyline quantification	Proteins	3312	7590	1331	12491	2545	5010	1367	8352
	Peptides	5343	9643	1473	29791	4667	6091	1710	18415
After removal of repeated peptides	Proteins	817	1966	364	3659	968	1813	551	3008
	Peptides	1121	2310	397	7056	1575	2159	623	6473

Supplemental Figure Legends

Supplemental Figure S1: Comparison of cleavage sites from TAILS within an alignment of Enterovirus B polyprotein sequences. The amino acid sequence of select Enterovirus B species' polyprotein were aligned using Muscle 3.8.425. Black arrows indicate the P1|P1' sites derived from the peptides in Figure 2, while grey arrows denote known viral cleavage sites which weren't detected. The viral polyproteins used in this analysis are as follows: Echovirus E18 E18/USA/9S8/2012 (WHA31300), Enterovirus B79 NH95-0601 (BAG70421), Enterovirus B86 BAN00-10354 (AAX47040), Echovirus E26 Coronel (AAQ73087), Echovirus E17 CHHE-29 (AAQ73080), Enterovirus B97 DT94-0227 (BAG70422), Echovirus E27 Bacon (AAQ73088), Echovirus E2 USA/2013-19511 (APA32000), Echovirus E15 CH 96-51 (AAQ73078), Echovirus E9 JH-983/2016 (WBY69253), Echovirus E14 Tow (AAQ73077), Echovirus E16 Harrington (AAQ73079), Echovirus E5 Kor-6-ECV5-253cn (ADO32836), Echovirus E31 Caldwell (AAQ73091), Echovirus E6 (APW91138), Coxsackievirus A9 BUCT01 (UJG81396), Enterovirus B107 TN94-0349 (BAG70420), Echovirus E3 123-R2 (QJR83058), Echovirus E12 K1529/YN/CHN/2013 (AWX63812), Echovirus E32 PR-10 (AAQ73092), Enterovirus B101 CIV03-10361 (AAX47044), Enterovirus B111 Q0011/XZ/CHN/2000 (AHE93351), Echovirus E7 HU/JH28/AM/BRA (WAB24477), Echovirus E19 NGR_2014 (AXQ00219), Echovirus E11 E11-PLA-NECK-23-E0-62-P1-FRA23 (WIL60174), Echovirus E25 XM0297 (AJR20996), Enterovirus B73 CA55-1988 (AAK13071), Echovirus E30 (ABF82247), Echovirus E21 Farina (AAQ73084), Echovirus E29 BRA/PA-29 (QHX99541), Echovirus E33 YNK35/CHN/2013 (AOW41918), Echovirus E1 Echo1_INMI1 (AVI00995), Echovirus E4 RL2017 (WBO25913), Echovirus E24 DeCamp (AAQ73085), Echovirus E20 KM-EV20-2010 (AHY03306), Enterovirus B75 Y26/XZ/CHN/2007 (QOW02255), Enterovirus B69 Toluca-1 (AAQ73097), Echovirus E13 Del Carmen (AAQ73076), Coxsackievirus B2 KOR-4-279 (ABM53474), Coxsackievirus B4 E2 (AAL37156), Coxsackievirus B5 18051/NX/CHN/2018 (WGZ82022), Coxsackievirus B1 KM64/YN/CHN/2019 (WIW72429), Coxsackievirus B6 Schmitt (AAF12719), Coxsackievirus B3 strain Nancy (AFS18536), Coxsackievirus B3 Kandolf (AAA42931).

Supplemental Figure S2: Locations of cleavages sites in select structures of Coxsackievirus B3 proteins. The P1 and P1' amino acids detected/inferred from the TAILS data are indicated on the protein structures (retrieved from the RCSB Protein Data Bank), represented either as sticks (only detected in HeLa samples), ball and sticks (only detected in HL1 samples) or spheres (detected in both HeLa and HL1 samples). Sites within 5 amino acids of the protein N-terminus were not indicated. Those detected only in the HeLa samples. Sites where the P1 site is an Arginine is coloured as heteroatoms. A & B) Two views of the Coxsackievirus B3 (strain Woodruff) coat protein (1COV) where the sites are coloured by capsid protein, with VP1 as grey, VP2 as teal, VP3 as light green and VP4 as forest green. C) The predicted cleavage sites on the Coxsackievirus B3 (strain Nancy) proteinase 3C (2VB0) and D) Coxsackievirus B3 Polymerase - F364I mutant (4ZP9) are coloured in purple.

Supplemental Figure S3: QQplot: Quantile-quantile plots of the H/L ratios of peptides identified in HeLa and HL1 samples. Peptides and consequently substrates outside of the linear range were identified as probable substrates in the two HeLa (A, B) and two HL1 (C,D) datasets.

Supplemental Figure S4: Substrate comparison: Comparisons of proteins and peptides found in HeLa and HL1 datasets. Venn diagrams of proteins/substrates (A) and peptides (D) that were identified in CVB3-infected HeLa and HL1 outside the linear range. Breakdown of positive H/L ratio substrates (B) and peptides (E), and negative H/L ratios of substrates (C) and peptides (F) identified from both HeLa and HL1 samples. G and H   Four factor venn diagrams of substrates (G) and peptides (H) indicating peptides that were unique and shared between positive and negative H/L ratio identified peptides between both HeLa and HL1 datasets.

Supplemental Figure S5: Network KEGG pathway analysis of A) high or B) low H/L ratio TAILS protein substrates from CVB3-infected HeLa cells.

Supplemental Figure S6: Network KEGG pathway analysis of A) high or B) low H/L ratio TAILS protein substrates from CVB3-infected HLI cells

Supplemental Figure S7: Immunoblotting of indicated proteins in CVB3-infected HLI (MOI 80). Representative immunoblots from at least two independent experiments.

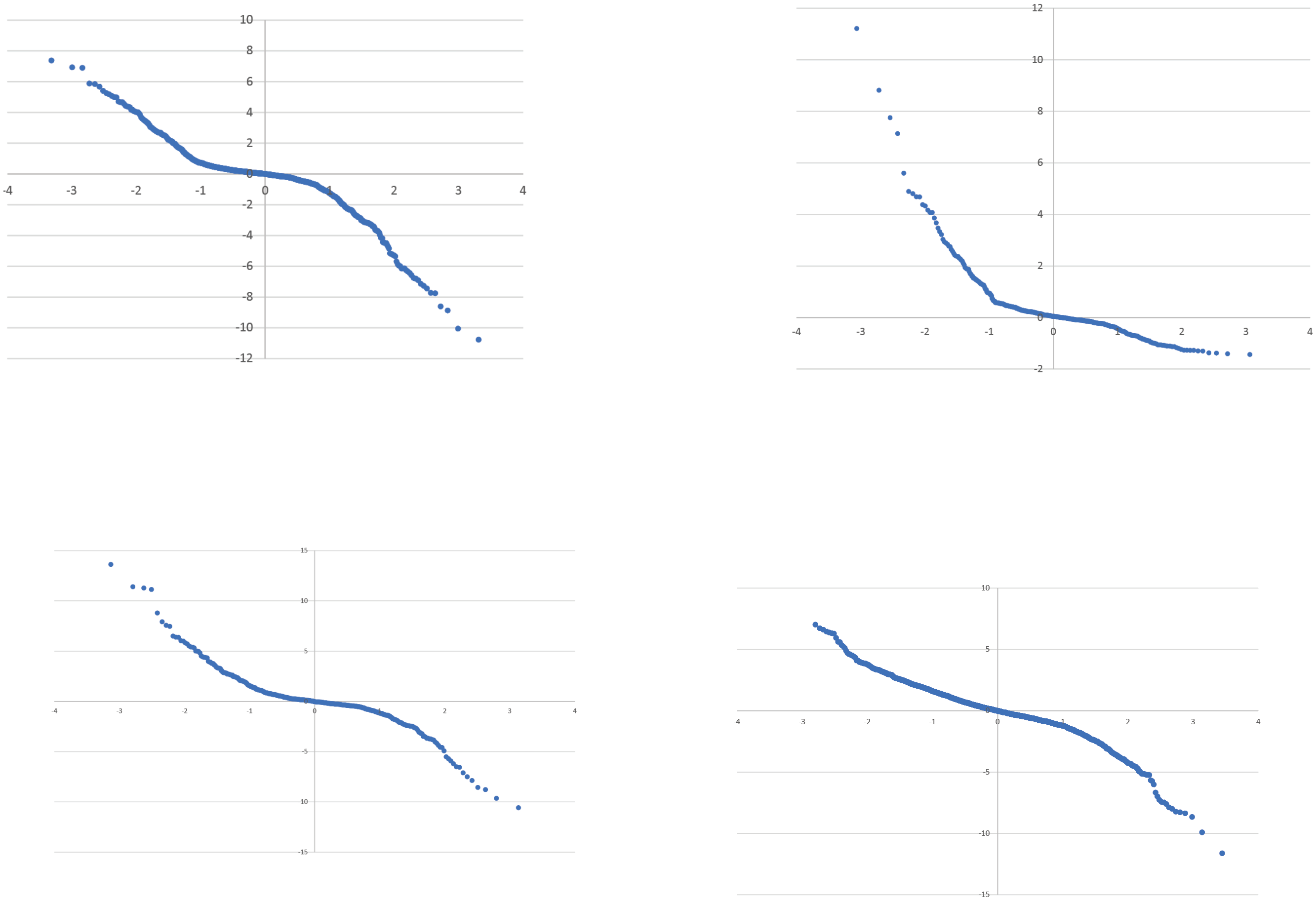

Supplemental Figure S3: Quantile-quantile plots of the H/L ratios of peptides identified in Hela and HL1 samples. Peptides and consequently substrates outside of the linear range were identified as probable substrates in the two Hela (A, B) and two HL1 (C,D) datasets.

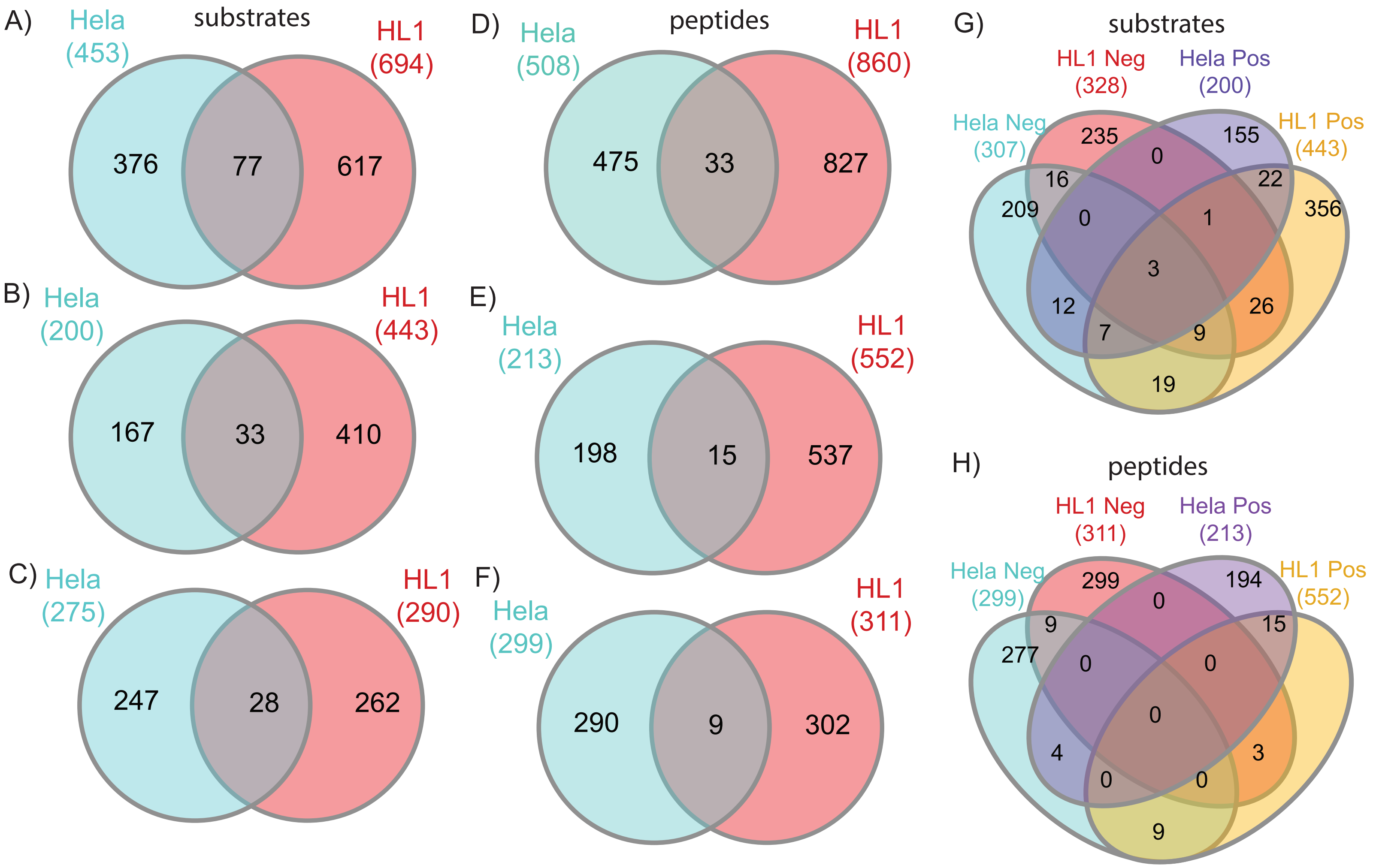

Figure S4 Venn diagrams of TAILS identified proteins/substrates (A) and peptides (D) that were identified in CVB3-infected Hela and HL1 outside the linear range. Breakdown of positive H/L ratio substrates (B) and peptides (E), and negative H/L ratios of substrates (C) and peptides (F) identified from both Hela and HL1 samples. G and H Four factor venn diagrams of Substrates (G) and peptides (H) indicating peptides that were unique and shared between positive and negative H/L ratio identified peptides between both Hela and HL1 datasets.

Vlok_Figure S5

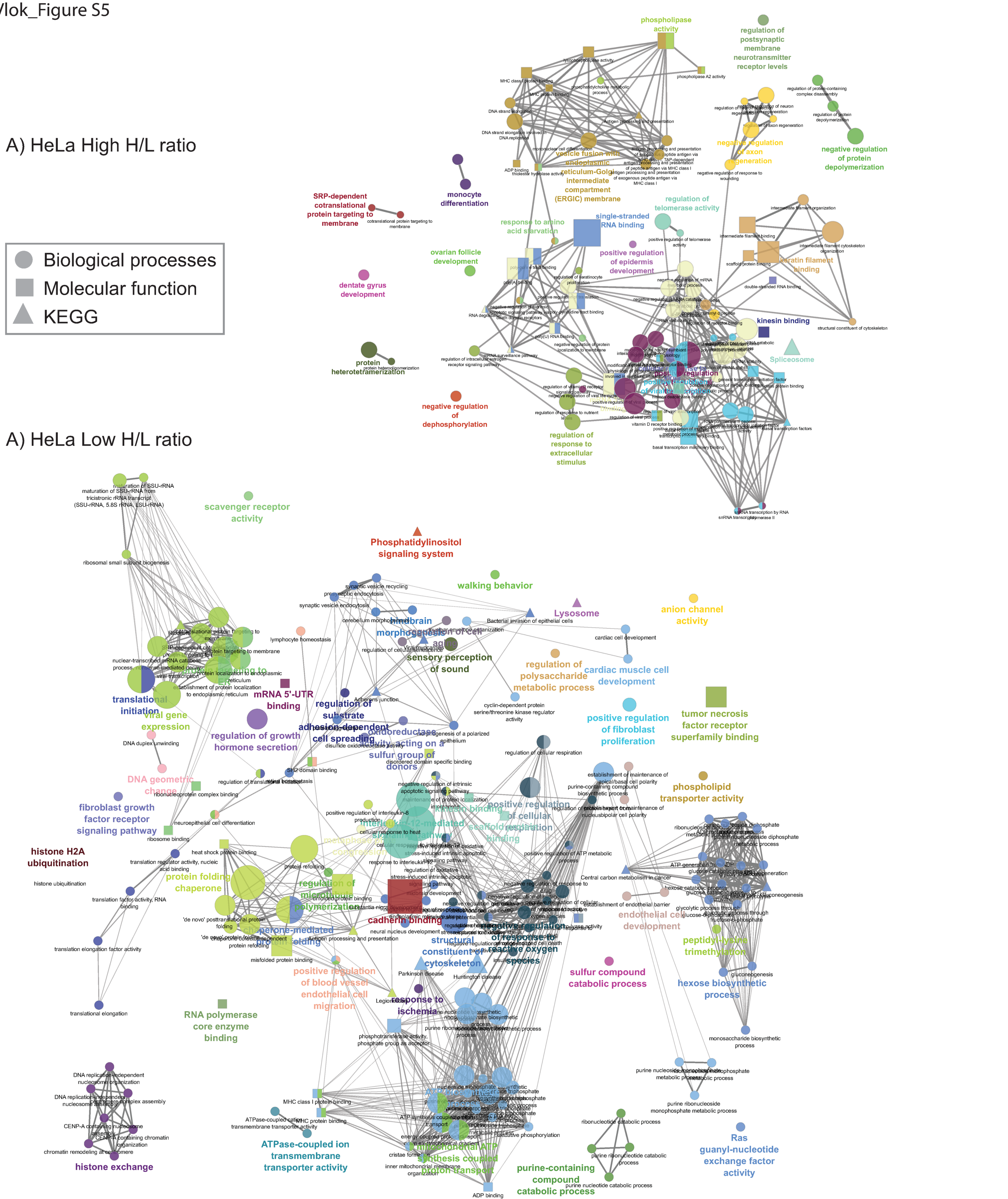

Vlok_Figure S6

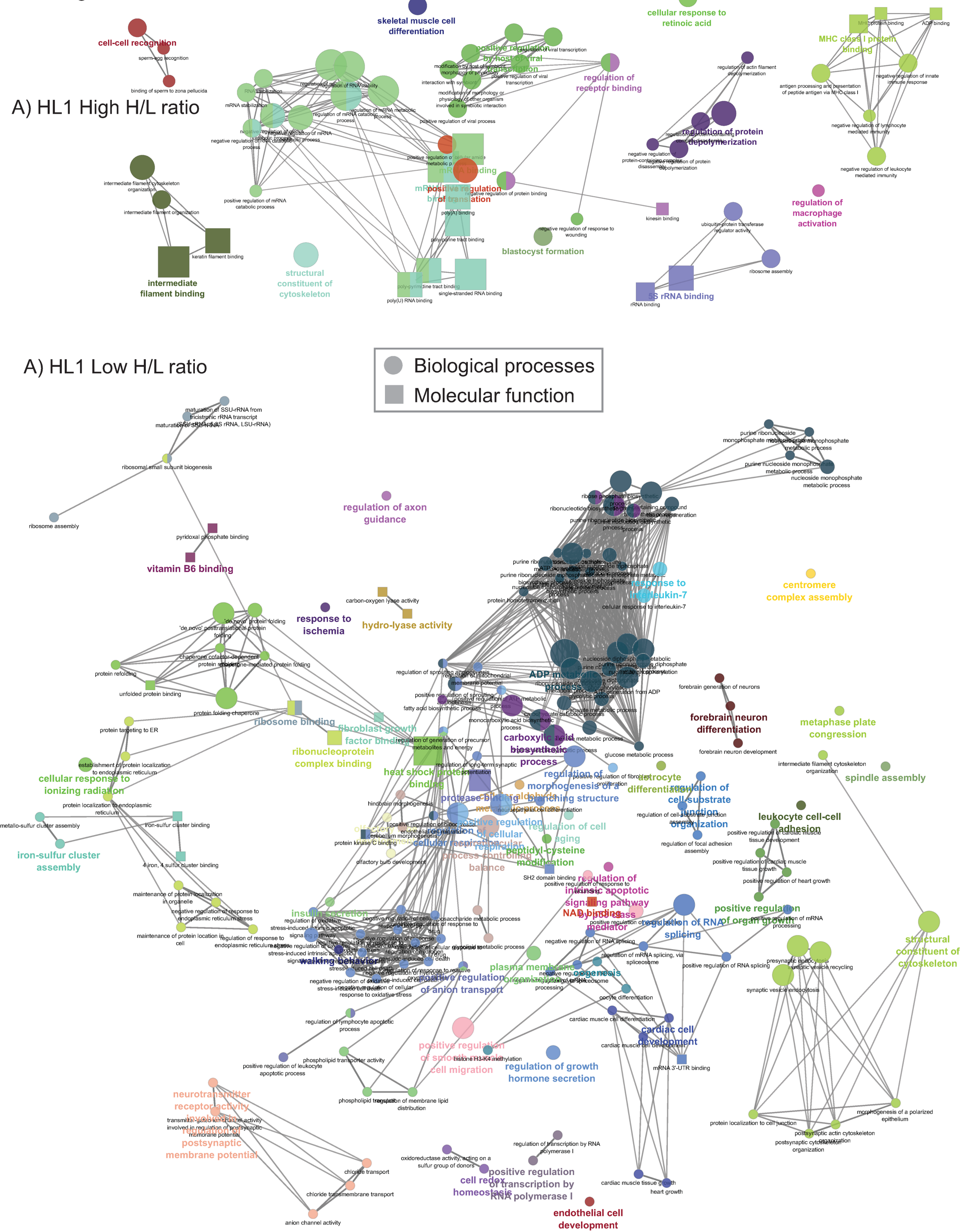

Supplemental Figure S7_Vlok et al

C HLI cells

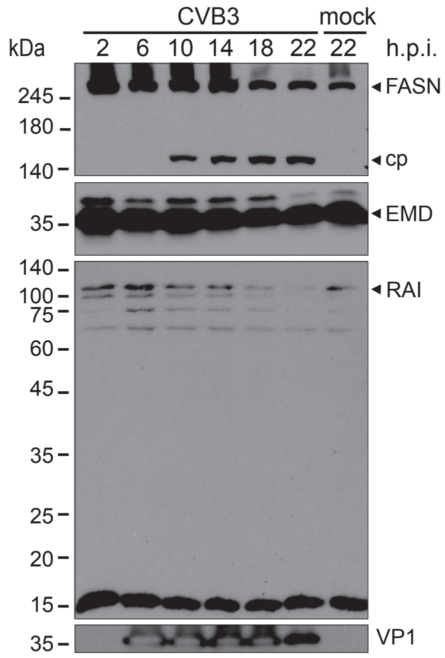

Supplement: Supplemental material — Table S1; Fig. S3 to S7. [file jvi.00498-24-s0003.pdf]
